# Supplementary material for: Microbial decomposition of biodegradable plastics on the deep-sea floor
Source: Nat Commun. 2024 Jan 26;15:568. doi: 10.1038/s41467-023-44368-8 (PMC10817984; doi:10.1038/s41467-023-44368-8)
Supplement: Supplementary file 1 — Supplementary Information [file 41467_2023_44368_MOESM1_ESM.pdf]

Supplementary Information

Microbial Decomposition of Biodegradable Plastics on the Deep-sea Floor

Taku Omura[1], Noriyuki Isobe[2], Takamasa Miura[3], Shun'ichi Ishii[4], Mihoko Mori[3], Yoshiyuki Ishitani[4], Satoshi Kimura[1], Kohei Hidaka[3], Katsuya Komiyama[1], Miwa Suzuki[5], Ken-ichi Kasuya[5,6], Hidetaka Nomaki[4], Ryota Nakajima[7], Masashi Tsuchiya[7], Shinsuke Kawagucci[7], Hiroyuki Mori[8], Atsuyoshi Nakayama[9], Masao Kunioka[10], Kei Kamino[3] and Tadahisa Iwata[1]*

[1] Laboratory of Science of Polymeric Materials, Department of Biomaterial Sciences, Graduate School of Agricultural and Life Sciences, The University of Tokyo, 1-1-1 Yayoi, Bunkyo-ku, Tokyo 113-8657, Japan

[2] Research Institute for Marine Resources Utilization, Japan Agency for Marine-Earth Science and Technology (JAMSTEC), 2-15 Natsushima-cho, Yokosuka, Kanagawa 237-0061, Japan

[3] Biological Resource Center, National Institute of Technology and Evaluation (NBRC), 2-5-8 Kazusakamatari, Kisarazu, Chiba 292-0818, Japan

[4] Institute for Extra-cutting-edge Science and Technology Avant-garde Research (X-STAR), Japan Agency for Marine-Earth Science and Technology (JAMSTEC), 2-15 Natsushima-cho, Yokosuka, Kanagawa 237-0061, Japan

[5] Gunma University Center for Food Science and Wellness (GUCFW), Maebashi, Gunma 371-8510, Japan

[6] Green Polymer Research Laboratory, Graduate School of Science and Technology, Gunma University, Kiryu, Gunma 376-8515, Japan

[7] Research Institute for Global Change (RIGC), Japan Agency for Marine-Earth Science and Technology (JAMSTEC), 2-15 Natsushima-cho, Yokosuka, Kanagawa 237-0061, Japan

[8] Japan BioPlastics Association (JBPA), 5-11 Nihonbashi Hakozaki-cho, Chuo-ku, Tokyo 103-0015, Japan

[9] Biomedical Research Institute, National Institute of Advanced Industrial Science and Technology (AIST), 1-8-31 Midorigaoka, Ikeda, Osaka 563-8577, Japan

[10] Standardization Promotion Office, National Institute of Advanced Industrial Science and Technology (AIST), 1-1-1 Umezono, Tsukuba, Ibaraki 305-8560, Japan

*All correspondence should be addressed to:
Tadahisa Iwata, atiwata@g.ecc.u-tokyo.ac.jp, +81-3-5841-5266

Supplementary Figures

Supplementary Fig. 1. Chemical structure of PHA, biodegradable polyesters, polysaccharide esters derivatives and non-biodegradable common plastics.

Supplementary Fig. 2. Deployment and recovery set-up at PJM, BHT, BMS, BMJ, and AKR

Supplementary Fig. 3. Deployment and recovery set-up at AMN

Supplementary Fig. 4. Research vehicles employed in the deployment and recovery of samples

Supplementary Fig. 5. Photographs of film samples, graphs of weight loss and film thickness loss, and surface SEM images before and after decomposition for a, PHB and b, PHBV.

Supplementary Fig. 6. Photographs of film samples, graphs of weight loss and film thickness loss, and surface SEM images before and after decomposition for c, P3HB4HB and d, PCL.

Supplementary Fig. 7. Photographs of film samples, graphs of weight loss and film thickness loss, and surface SEM images before and after decomposition for e, PBS and f, PBAT.

Supplementary Fig. 8. Photographs of film samples, graphs of weight loss and film thickness loss, and surface SEM images before and after decomposition for g, PE and h, PS.

Supplementary Fig. 9. Photographs of film samples, graphs of weight loss and film thickness loss, and surface SEM images before and after decomposition for i, PET.

Supplementary Fig. 10. SEM images of four PHA films placed on the BHT site for three months. Color 3D maps of surface roughness and attached micro-organism images are shown in pairs.

Supplementary Fig. 11. SEM images of five types of biodegradable polyesters films placed on the BHT site for three months. Surface roughness images by color 3D mapping and microstructures of attached bacteria are shown in pairs (Note that the 3D map magnification was larger than that of the PHA films because of their slow biodegradation).

Supplementary Fig. 12. SEM images of four types of common plastic films placed on the BHT site for three months. Surface roughness images by color 3D mapping and microstructures of attached bacteria are shown in pairs (Note that the 3D map magnification was larger than that of the PHA films because of their slow biodegradation).

Supplementary Fig. 13. 16S rRNA gene-based prokaryotic community composition of biofilms from PHA, biodegradable polyesters, and non-biodegradable common plastics, at the family-level.

Supplementary Fig. 14. Metagenome-based microbial community compositions of plastisphere established on various plastics at the sea.

Supplementary Fig. 15. Metagenomic assembly of deep-seawater and sediment microbiomes near plastic degradation test at site BHT.

Supplementary Fig. 16. Metagenome-based microbial community compositions of plastisphere compared with surrounding deep seawater and sediment under the plastic chamber at site BHT.

Supplementary Fig. 17. Comparison of microbial community compositions among plastispheres, the deep-sea microbiome, and sediments at site BHT.

Supplementary Fig. 18. Polysaccharide-associated bacterial communities vary by degree of acetylation, site, and immersion period.

Supplementary Fig. 19. 16S rRNA gene-based prokaryotic community composition of polysaccharides and common plastics at the family level.

Supplementary Fig. 20. Comparison of microbial community compositions of the plastispheres based on a multidimensional scale plot.

Supplementary Fig. 21. Phylogenetic tree of single-copy housekeeping gene, ribosomal protein S3, RpsC, identified in plastisphere metagenomes at the sea.

Supplementary Fig. 22. Shade plot illustrating the relative abundances (%) of plastic-specific amplicon sequence variants (ASVs) at different sites and periods of immersion

Supplementary Fig. 23. Heatmap of the 50 most abundant ASVs in biofilms obtained from all polysaccharides and non-biodegradable common plastics.

Supplementary Fig. 24. Shade plot illustrating the relative abundances of plastic specific metagenome-assembled genomes (MAGs) at different sites and periods of immersion.

Supplementary Fig. 25. Additional information for MAGs of dominant microbes within plastisphere established on biodegradable polyesters at the sea.

Supplementary Fig. 26. Genome clustering from metagenome sequences of plastisphere for PHB and PHBV established under oceanic condition.

Supplementary Fig. 27. Genome clustering from metagenome sequences of plastisphere for PHBH and P3HB4HB established under oceanic condition.

Supplementary Fig. 28. Genome clustering from metagenome sequences of plastisphere for PBAT and PBSA established under oceanic condition.

Supplementary Fig. 29. Genome clustering from metagenome sequences of plastisphere for PCL and PLLA established under oceanic condition.

Supplementary Fig. 30. Genome clustering from metagenome sequences of plastisphere for PET and PE established under oceanic condition.

Supplementary Fig. 31. Genome clustering from metagenome sequences of plastisphere for PS and PP established under oceanic condition.

Supplementary Tables

Supplementary Table 1. Detail of samples placed for 3 months at BHT (BHT03).

Supplementary Table 2. Detail of samples placed for 8 months at BHT (BHT08).

Supplementary Table 3. Biodegradation rate of injection-molded plastic samples.

Supplementary Table 4. Biodegradation rates of various melt-pressed PHA and biodegradable polyester films.

Supplementary Table 5. Detail of samples placed for 2 months at PJM (PJM02).

Supplementary Table 6. Detail of samples placed for 5 months at BMS (BMS05).

Supplementary Table 7. The weight-loss of polysaccharide esters derivatives films.

Supplementary Table 8. Stats for metagenomic assemblies of plastisphere at the sea.

Supplementary Table 9. Minimum information of metagenome-assembled genome (MiMAG) and GBDBtk taxonomy of MAGs.

Supplementary Table 10. Information of raw sequence data for 16S rRNA gene amplicon sequencing and metagenomic sequencing.

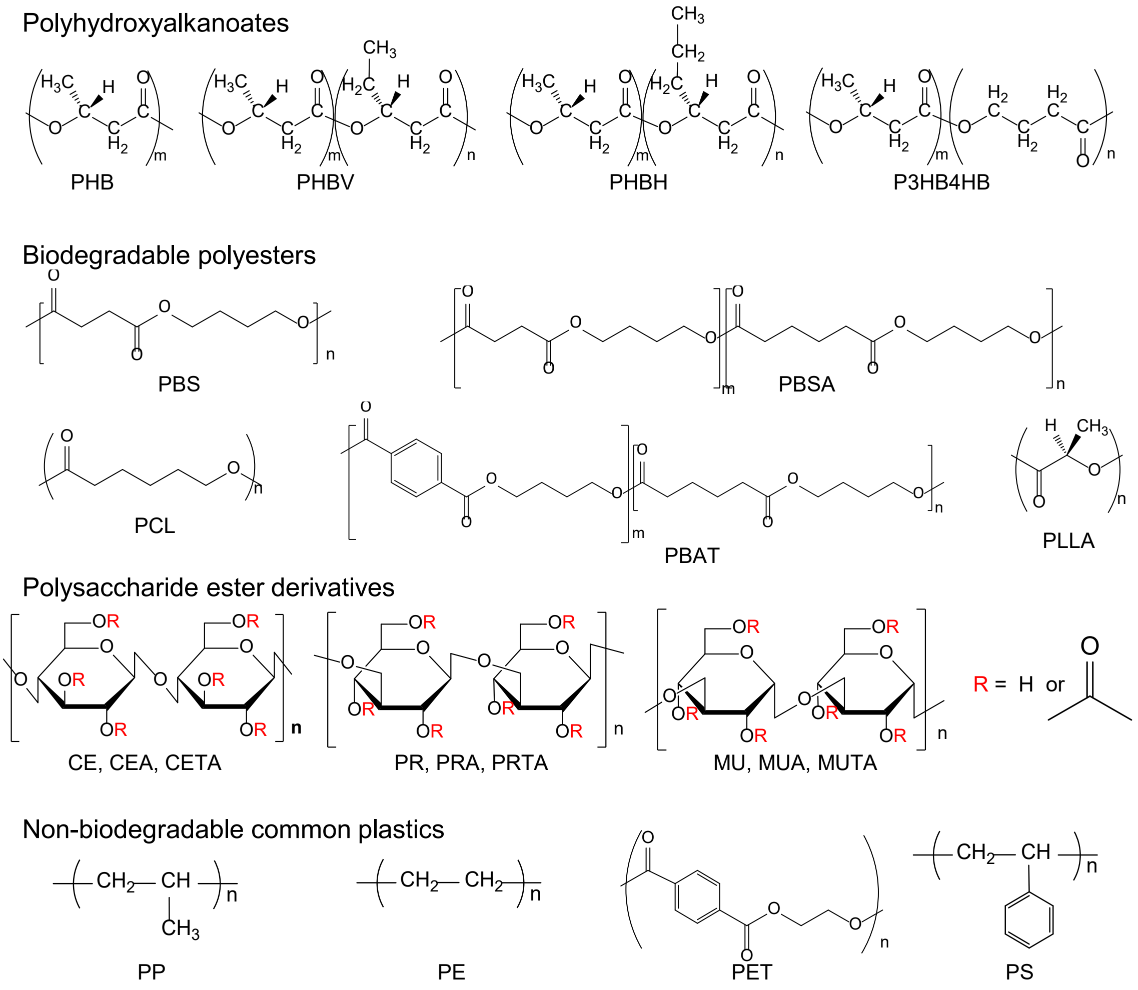

Supplementary Fig. 1 | Chemical structure of PHA, biodegradable polyesters, polysaccharide ester derivatives and non-biodegradable common plastics.

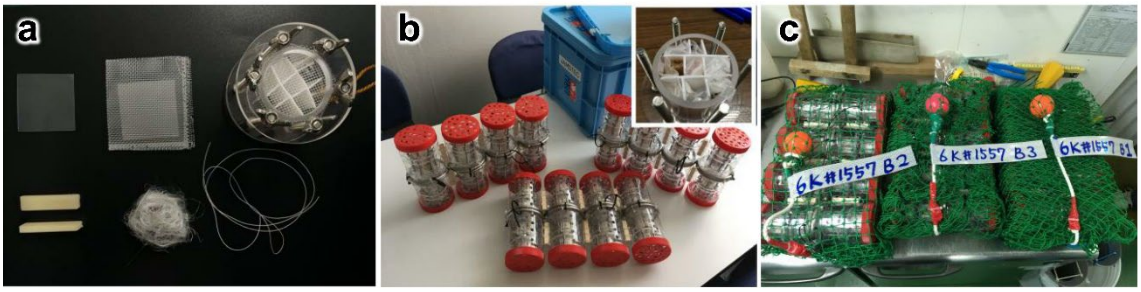

Supplementary Fig. 2 | Deployment and recovery set-up at PJM, BHT, BMS, BMJ, and AKR

a, sample specimen used for the on-site degradation tests at BHT, BMS, BMJ, and AKR, which were stored in the sample holder, **b,** sample chambers where the sample holder (inset) were stored and **c,** sample chambers covered with protective nets and tagged with buoy.

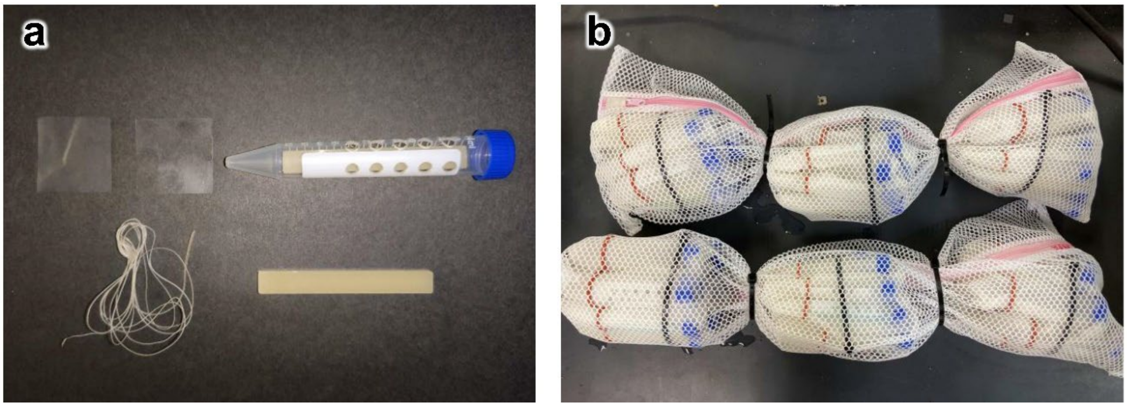

Supplementary Fig. 3 | Deployment and recovery set-up at AMN

a, sample specimen used for the on-site degradation tests at AMN, which were stored in the centrifuge tubes with holes used as a sample holder and **b,** sample holders covered with protective nets.

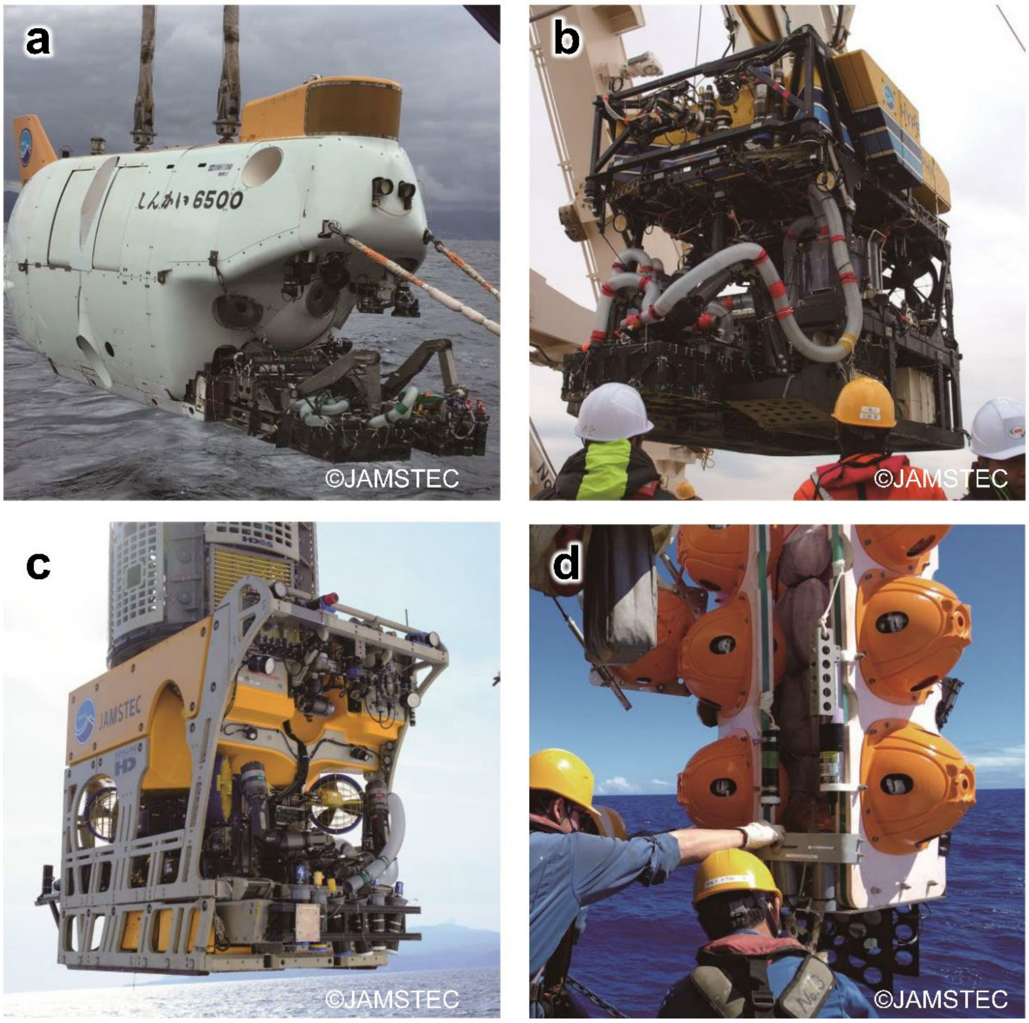

Supplementary Fig. 4 | Research vehicles employed in the deployment and recovery of samples

a, human occupied vehicle (HOV) *Shinkai 6500*, **b,** remotely operated vehicles (Hyper-Dolphin), **c,** remotely operated vehicles (Kaimei-ROV) and **d,** the free-fall-type deep-sea observatory lander system Edokko Mark 1 employed at AMN. The photos were taken by the authors of this work and are used in accordance with the data use policy of the Japan Agency for Marine Earth Science and Technology (https://www.jamstec.go.jp/e/database/data_policy.html).

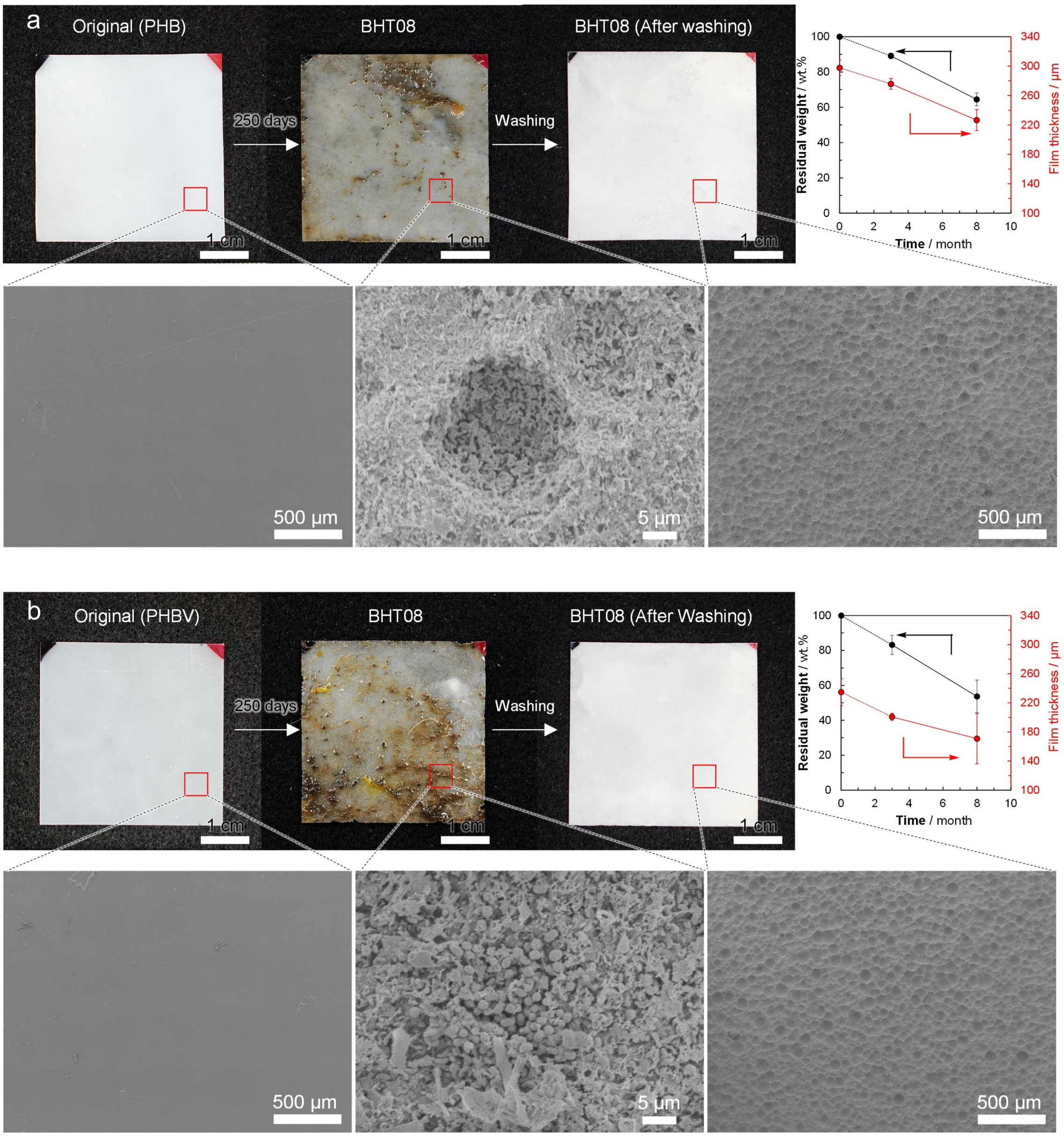

Supplementary Fig. 5 | Photographs of film samples, graphs of weight loss and film thickness loss, and surface SEM images before and after decomposition for a, poly[(*R*)-3-hydroxybutyrate] (PHB) and b, poly[(*R*)-3-hydroxybutyrate-*co*-12 mol%-(*R*)-3-hydroxyvalerate] (PHBV).

After 250 days of deployment at 855 m (the deep-sea floor) off Hatsushima Island (BHT08), the recovered films were ultrasonically washed to remove biofilm, vacuum dried, and then evaluated for weight loss (black line) and film thickness decrease (red line). Values are given as average of $n = 4$ independent samples with its standard deviation (Supplementary Data 1). The scanning electron microscope (SEM) images show a magnified view of the surface of each film. While the surface of the original sample was smooth, microorganisms accumulated on the surface of the BHT08 sample, and the surface of the film after the microorganisms were removed by ultrasonic washing (BHT08 After washing) showed roughness typically observed following microbial degradation. The micrographs are representative images from $n = 3$ independent samples with similar results. Source data are provided as a Source Data file.

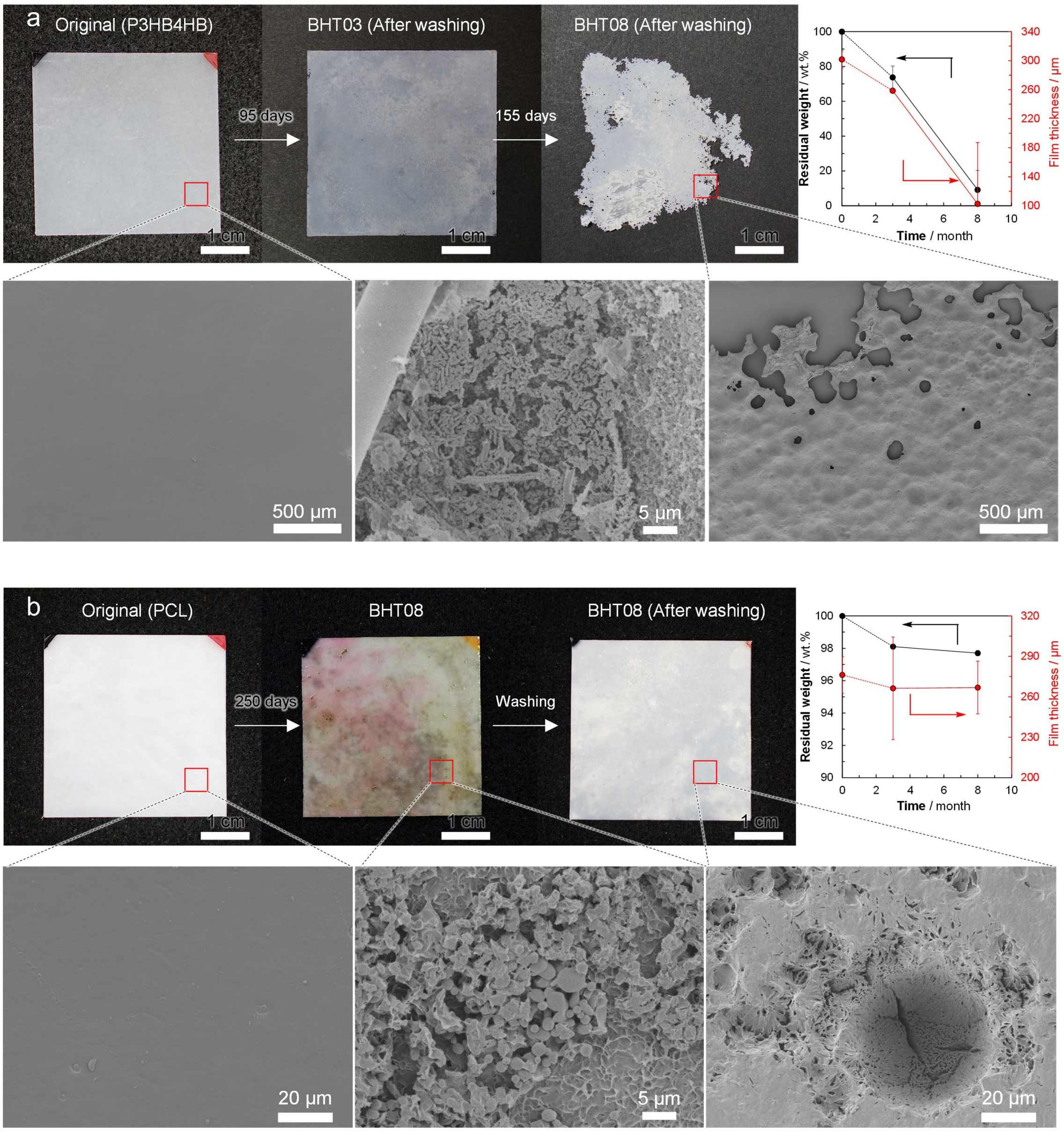

Supplementary Fig. 6 | Photographs of film samples, graphs of weight loss and film thickness loss, and surface SEM images before and after decomposition for a, poly[(*R*)-3-hydroxybutyrate-*co*-16 mol%-4-hydroxybutyrate] (P3HB4HB) and b, poly(ε-caprolactone) (PCL).

After 250 days of deployment at 855 m (the deep-sea floor) off Hatsushima Island (BHT08), the recovered films were ultrasonically washed to remove biofilm, vacuum dried, and then evaluated for weight loss (black line) and film thickness decrease (red line). Values are given as average of $n = 4$ independent samples with its standard deviation (Supplementary Data 1). The scanning electron microscope (SEM) images show a magnified view of the surface of each film. While the surface of the original sample was smooth, microorganisms accumulated on the surface of the BHT08 sample, and the surface of the film after the microorganisms were removed by ultrasonic washing (BHT08 After washing) showed roughness typically observed following microbial degradation. The micrographs are representative images from $n = 3$ independent samples with similar results. Source data are provided as a Source Data file.

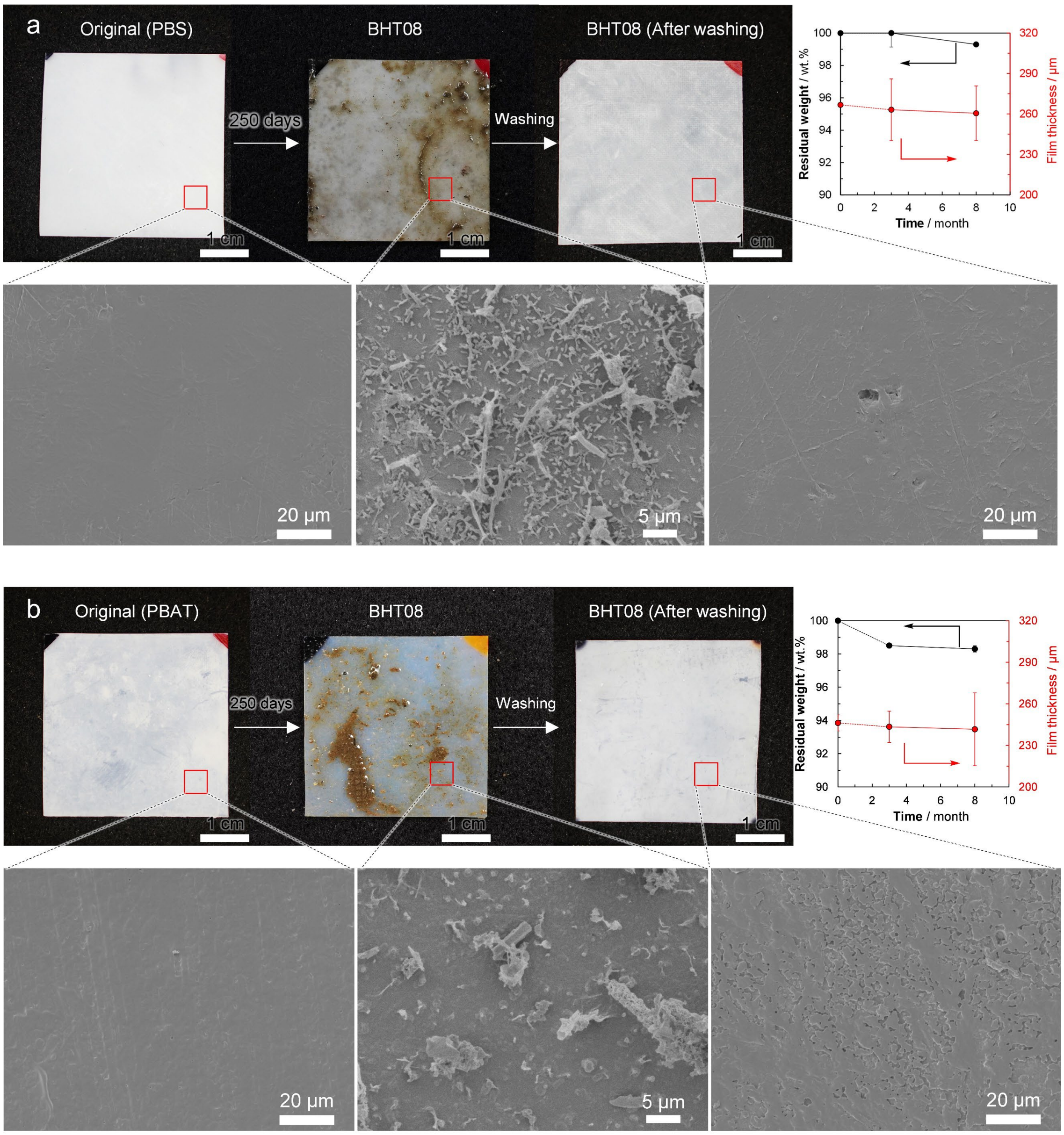

Supplementary Fig. 7 | Photographs of film samples, graphs of weight loss and film thickness loss, and surface SEM images before and after decomposition for a, poly(butylene succinate) (PBS) and b, poly(butylene adipate-*co*-terephthalate) (PBAT).

After 250 days of deployment at 855 m (the deep-sea floor) off Hatsushima Island (BHT08), the recovered films were ultrasonically washed to remove biofilm, vacuum dried, and then evaluated for weight loss (black line) and film thickness decrease (red line). Values are given as average of $n = 4$ independent samples with its standard deviation (Supplementary Data 1). The scanning electron microscope (SEM) images show a magnified view of the surface of each film. While the surface of the original sample was smooth, microorganisms accumulated on the surface of the BHT08 sample, and the surface of the film after the microorganisms were removed by ultrasonic washing (BHT08 After washing) showed roughness typically observed following microbial degradation. The micrographs are representative images from $n = 3$ independent samples with similar results. Source data are provided as a Source Data file.

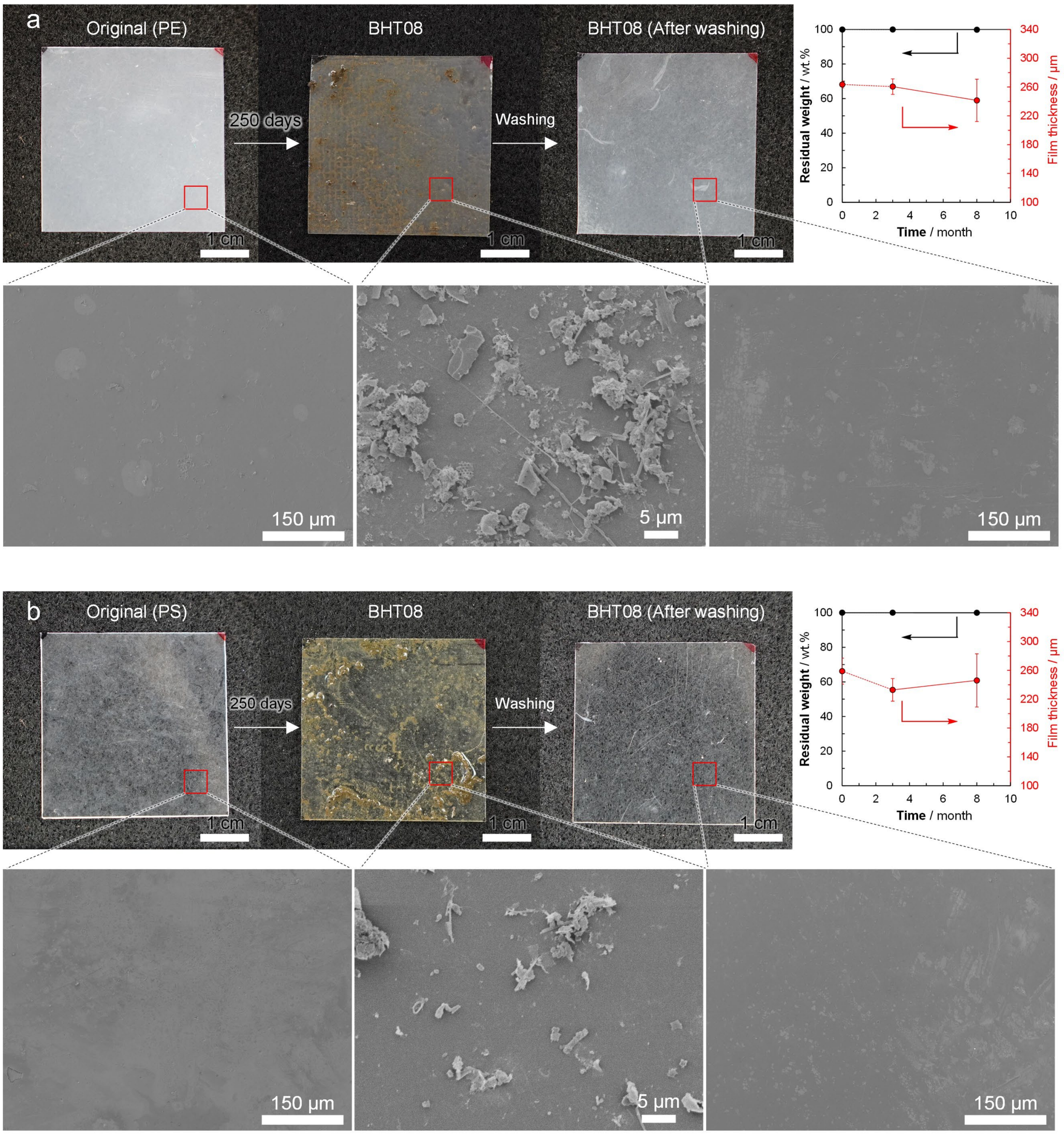

Supplementary Fig. 8 | Photographs of film samples, graphs of weight loss and film thickness loss, and surface SEM images before and after decomposition for a, low-density Polyethylene (PE) and b, polystyrene (PS).

After 250 days of deployment at 855 m (the deep-sea floor) off Hatsushima Island (BHT08), the recovered films were ultrasonically washed to remove biofilm, vacuum dried, and then evaluated for weight loss (black line) and film thickness decrease (red line). Values are given as average of $n = 2$ independent samples with its standard deviation (Supplementary Data 1). The scanning electron microscope (SEM) images show a magnified view of the surface of each film. The original surface of the common plastic was smooth like the biodegradable plastic; little adhesion was observed on BHT08; and even after the surface adhesion was removed by ultrasonic washing (BHT08 After washing), a smooth surface was observed, as for the original films. The micrographs are representative images from $n = 3$ independent samples with similar results. Source data are provided as a Source Data file.

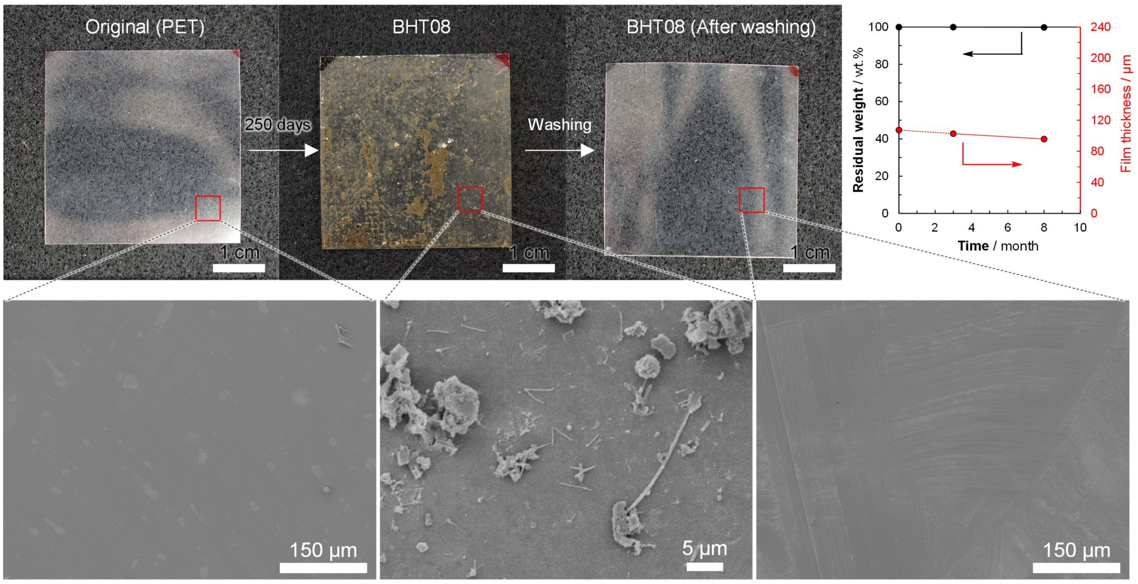

Supplementary Fig. 9 | Photographs of film samples, graphs of weight loss and film thickness loss, and surface SEM images before and after decomposition for polyethylene terephthalate (PET). After 250 days of deployment at 855 m (the deep-sea floor) off Hatsushima Island (BHT08), the recovered films were ultrasonically washed to remove biofilm, vacuum dried, and then evaluated for weight loss (black line) and film thickness decrease (red line). Values are given as average of $n = 2$ independent samples with its standard deviation (Supplementary Data 1). The scanning electron microscope (SEM) images show a magnified view of the surface of the film. The original surface was smooth like the biodegradable plastic; little adhesion was observed on BHT08; and even after the surface adhesion was removed by ultrasonic washing (BHT08 After washing), a smooth surface was observed, as for the original film. The micrographs are representative images from $n = 3$ independent samples with similar results. Source data are provided as a Source Data file.

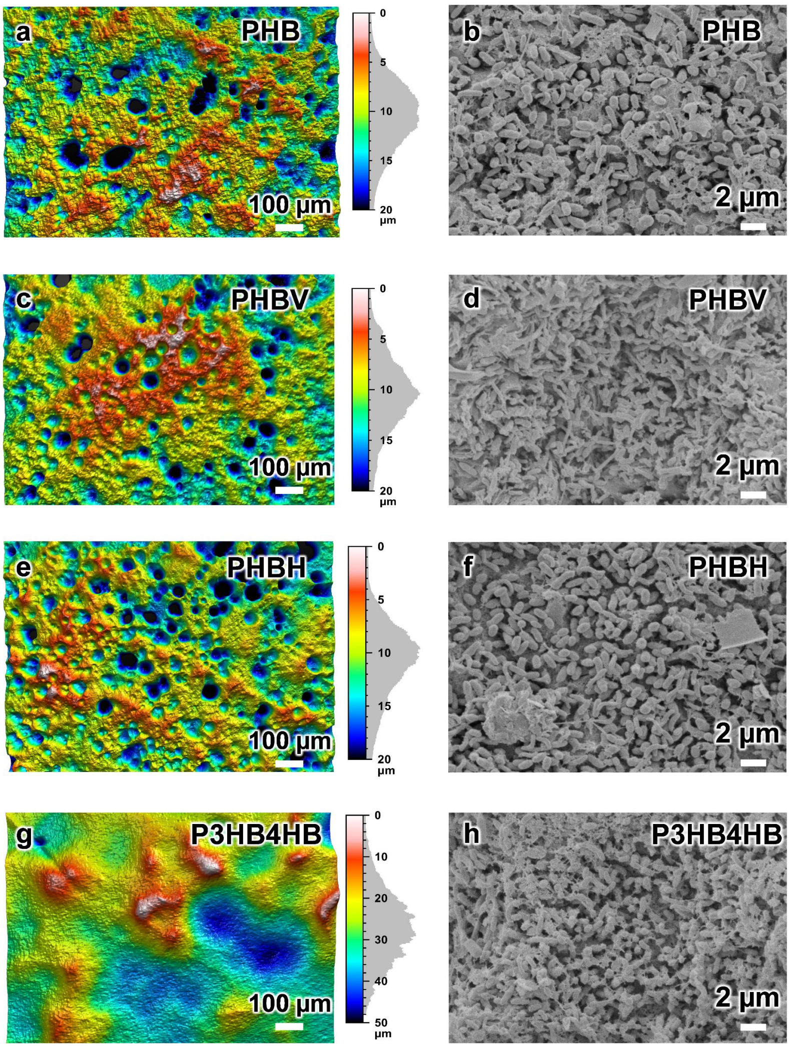

Supplementary Fig. 10 | SEM images of four PHA films placed on the BHT site for three months. Color 3D maps of surface roughness and attached micro-organism images are shown in pairs.
In the 3D maps of **a,** PHB, **c,** PHBV, and **e,** PHBH, numerous circular erosions with a diameter of 10-100 µm, with an average depth of 10 µm were observed. In the higher magnified images, numerous bacterial growths were observed in the erosion area **b, d, f** and **h**. These results strongly suggest that the erosions of the sample surface were caused by bacterial enzymatic biodegradation. The P3HB4HB was observed that the fastest weight loss ratio in deep sea. In the SEM observation, surface erosion of **g,** P3HB4HB films were progressed further than other PHA films. Large hole with several hundred µm diameter were observed where multiple holes were fused together, and the average depth of erosion was 30 µm (Note that the color scale of g, is shown at 50 µm). The micrographs are representative images from *n* = 3 independent samples with similar results. Source data are provided as a Source Data file.

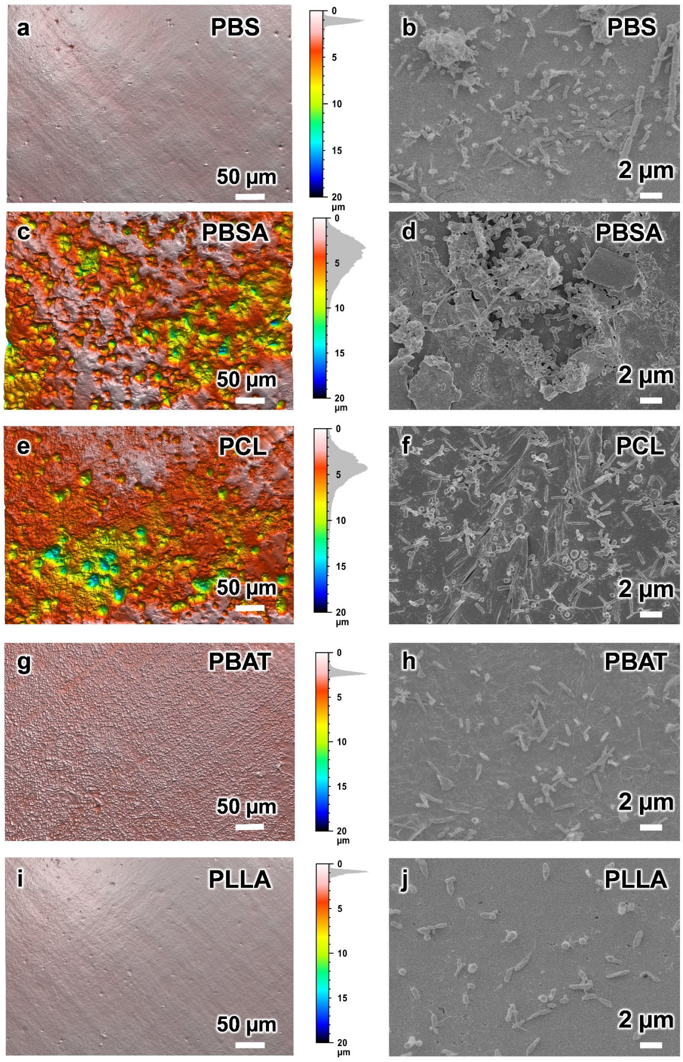

Supplementary Fig. 11 | SEM images of five types of biodegradable polyesters films placed on the BHT site for three months. Surface roughness images by color 3D mapping and microstructures of attached bacteria are shown in pairs (Note that the 3D map magnification was larger than that of the PHA films because of their slow biodegradation).

In observation of the biodegradable polyesters **a, b** PBS, **c, d** PBSA, **e, f** PCL, **g, h** PBAT and **i, j** PLLA, significant surface erosion was observed in **c** PBSA and **e** PCL, with showing circular erosions up to 50 µm in diameter and an average depth of 5 µm, respectively. Bacterial growth was observed in the magnified images **d, f**. In the **g** PBAT film showed fine erosion, suggesting that early stage of degradation. On the other hand, no surface erosion was observed in **a**, PBS and **i** PLLA, however, the amount of attached bacteria was clearly higher on the surface of PBS film than that of **i** PLLA. It suggests that the bacterial growth was still early stage. The micrographs are representative images from n = 3 independent samples with similar results. Source data are provided as a Source Data file.

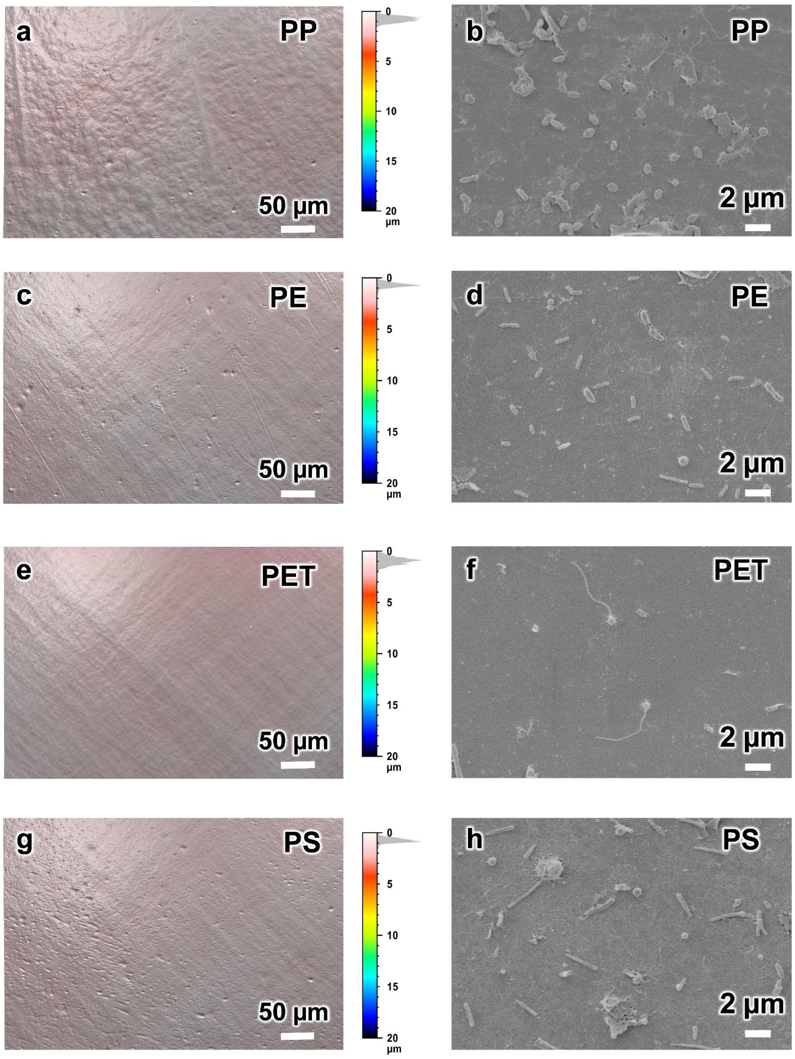

Supplementary Fig. 12 | SEM images of four types of common plastic films placed on the BHT site for three months. Surface roughness images by color 3D mapping and microstructures of attached bacteria are shown in pairs (Note that the 3D map magnification was larger than that of the PHA films because of their slow biodegradation).

No surface erosion was observed in any common plastics, and although bacteria were observed adhering on the surface, no signs of specific bacterial growth were observed **a-h**. The micrographs are representative images from $n = 3$ independent samples with similar results. Source data are provided as a Source Data file.

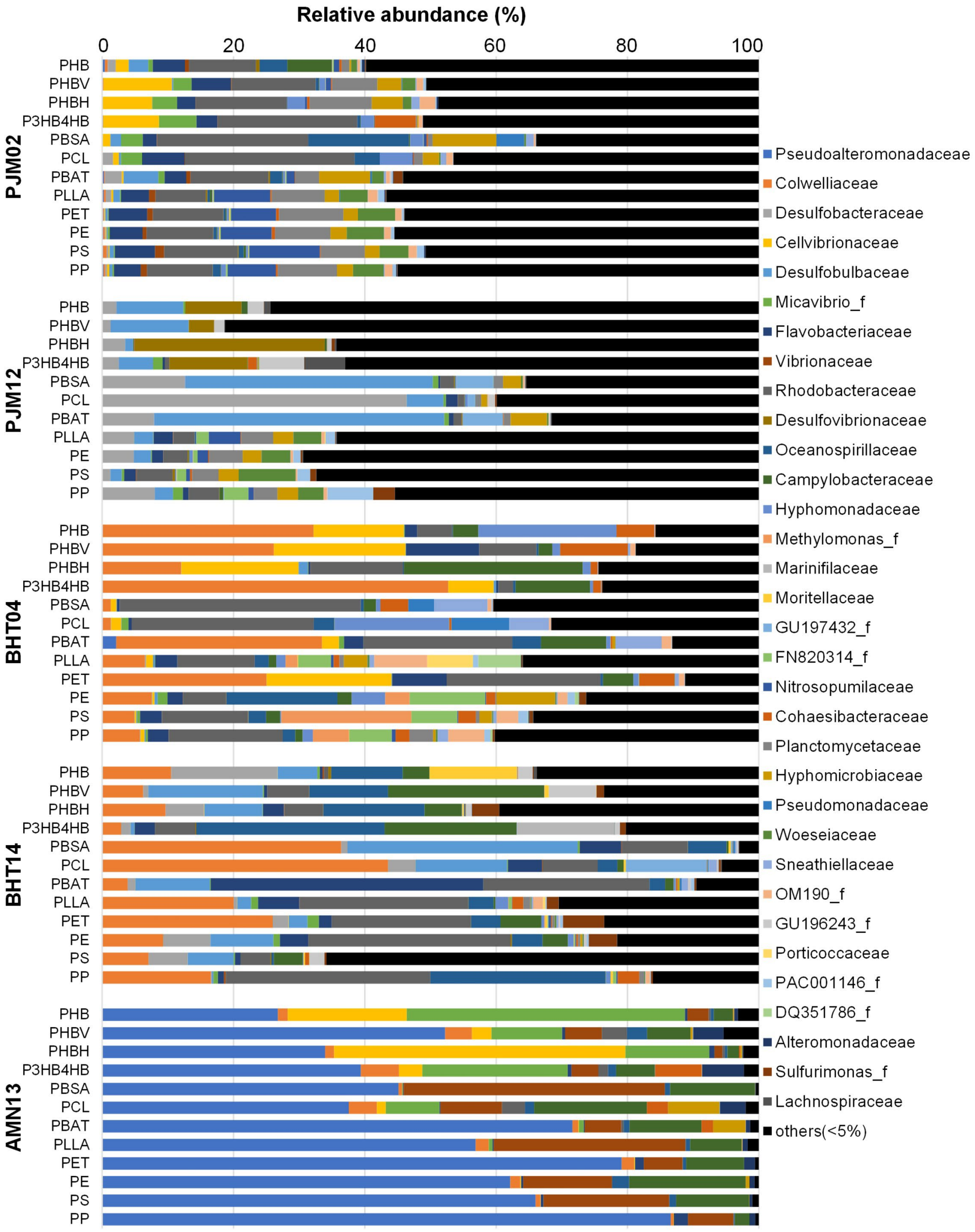

Supplementary Fig. 13 | 16S rRNA gene-based prokaryotic community composition of biofilms from PHA, biodegradable polyesters, and non-biodegradable common plastics, at the family-level. “Others” are families representing <5% relative abundance.

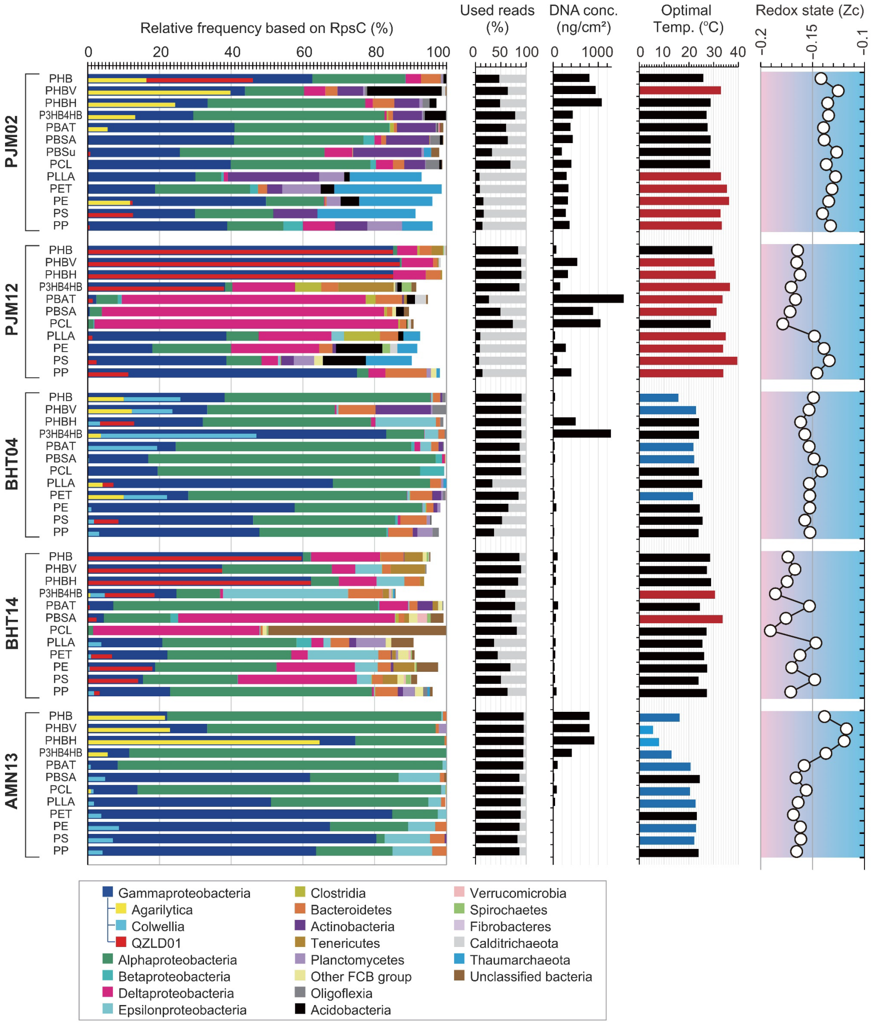

Supplementary Fig. 14 | Metagenome-based microbial community compositions of plastisphere established on various plastics at the sea.

Colored bar chart shows comparison of microbial community compositions based on Phylum or Class taxa of RpsC gene from the metagenomic analysis. As for *Gammaproteobacteria*, three main lower-level taxa are shown in lower bar. Usage of metagenomic raw reads for the *de novo* assembly was calculated after mapping them to the scaffolds, while DNA concentration per plastic surface area was determined after extracting DNA.

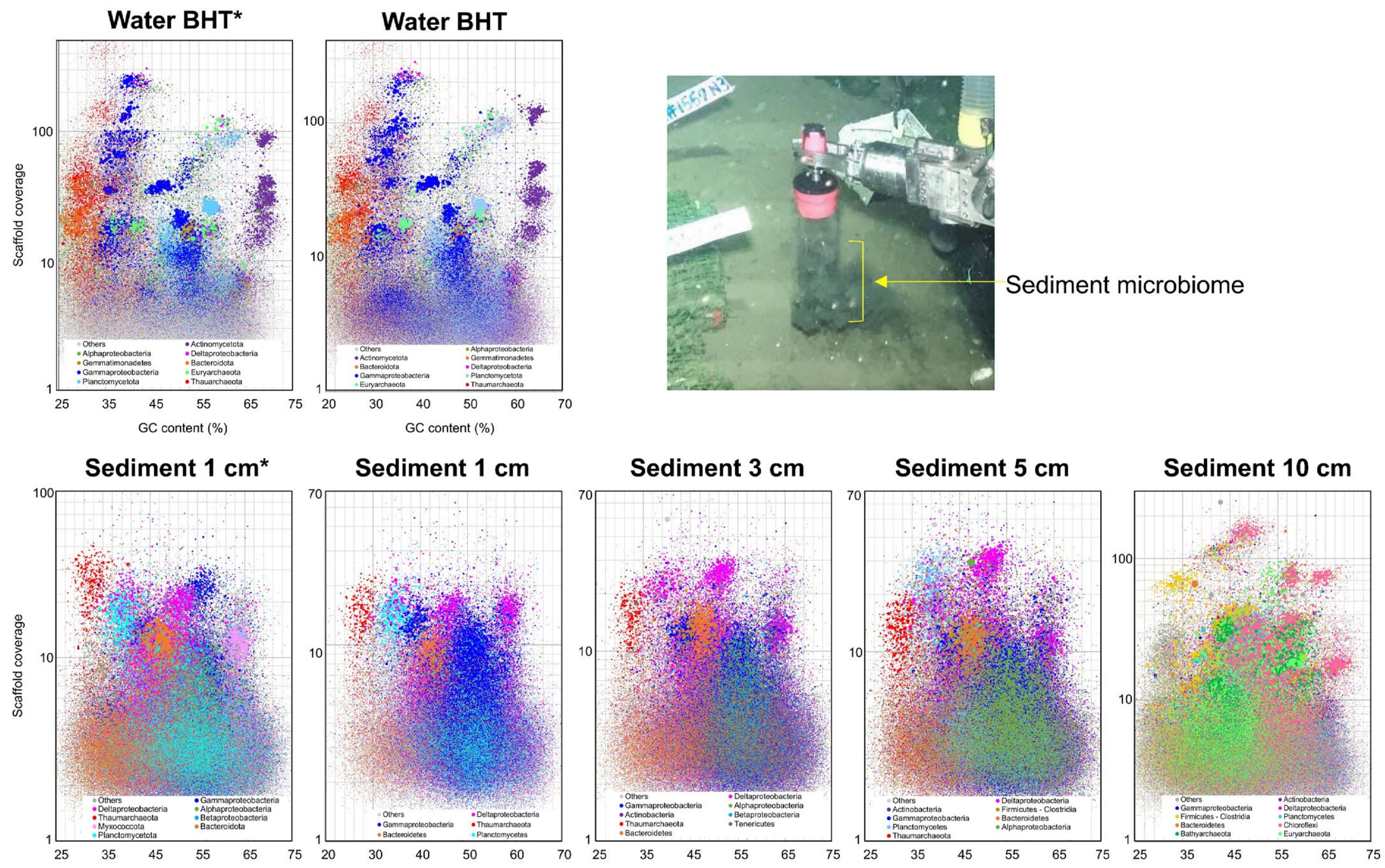

Supplementary Fig. 15 | Metagenomic assembly of deep-seawater and sediment microbiome near plastic degradation test at site BHT.

Genome clusters from the assembled scaffolds from metagenomic sequences were identified using the estimated taxonomic classification (color of dots), length (size of dots), G+C content (*x*-axis), and mean coverage of scaffold (*y*-axis). * indicates microbiome near plastic-containing chambers.

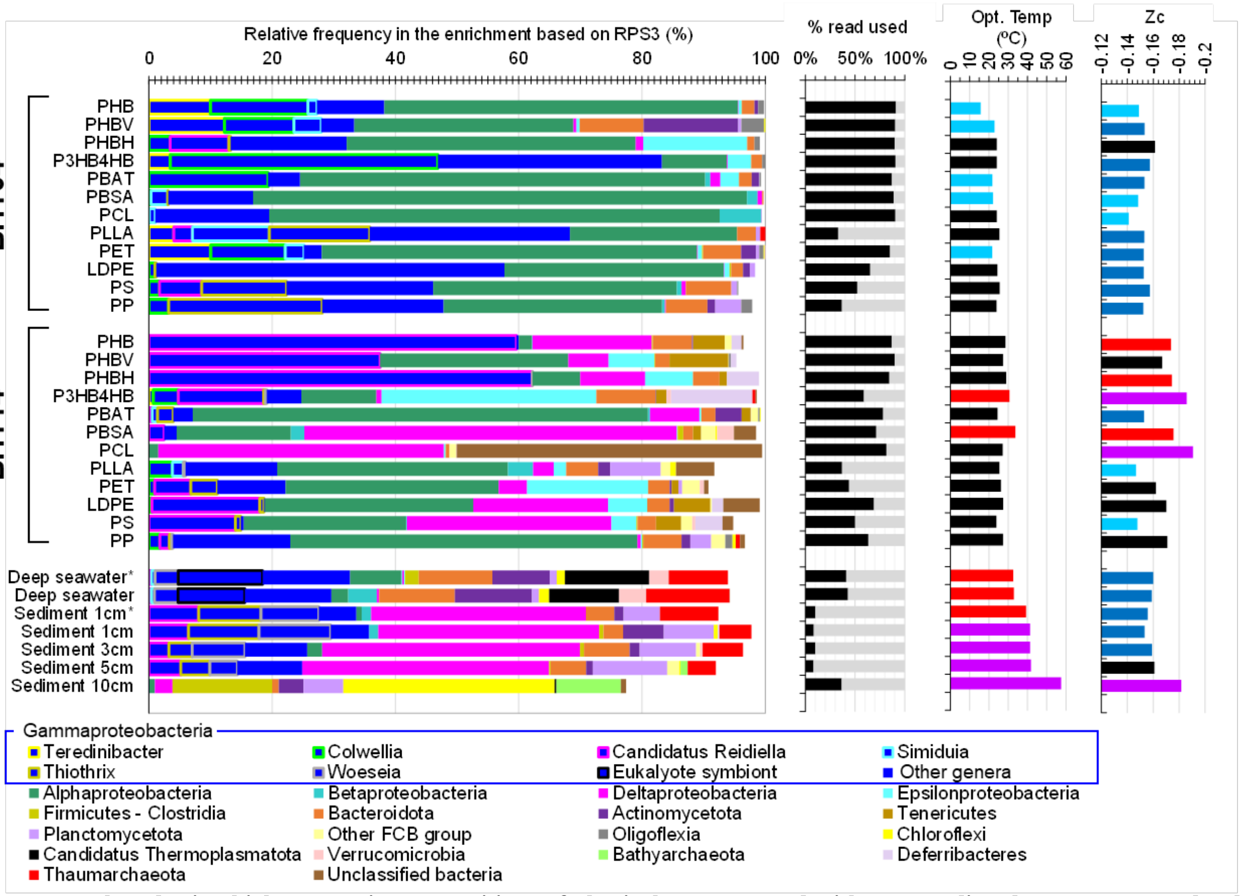

Supplementary Fig. 16 | Metagenome-based microbial community compositions of plastisphere compared with surrounding deep seawater and sediment under the plastic chamber at site BHT.

The bar chart shows comparison of microbial community compositions (phylum or class) based on RpsC gene sequences from the metagenomic analysis. For *Gammaproteobacteria* (blue bar), seven main genera are highlighted by different colors. Use of metagenomic raw reads for the *de novo* assembly, estimated optimal growth temperature of the taxon, and estimated redox state (Z_c) are shown as bar charts.

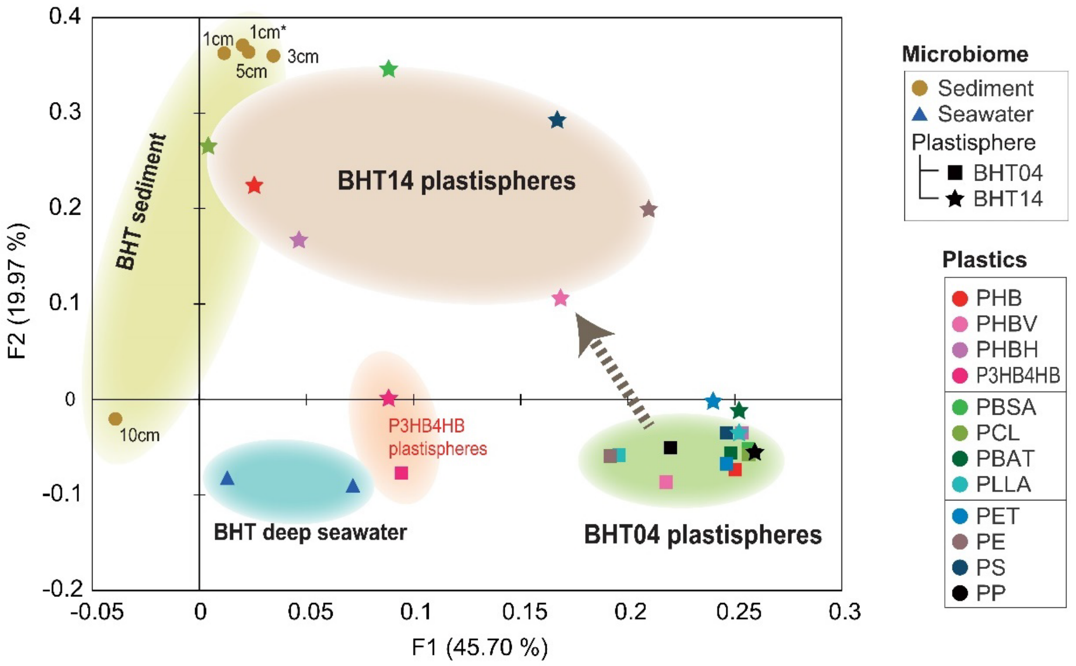

Supplementary Fig. 17 | Comparison of microbial community compositions among plastispheres, the deep-sea microbiome, and sediments at site BHT.

Principal component analysis was performed based on phylum-level microbial community composition as described in Supplementary Fig. 16 for the class *Gammaproteobacteria*.

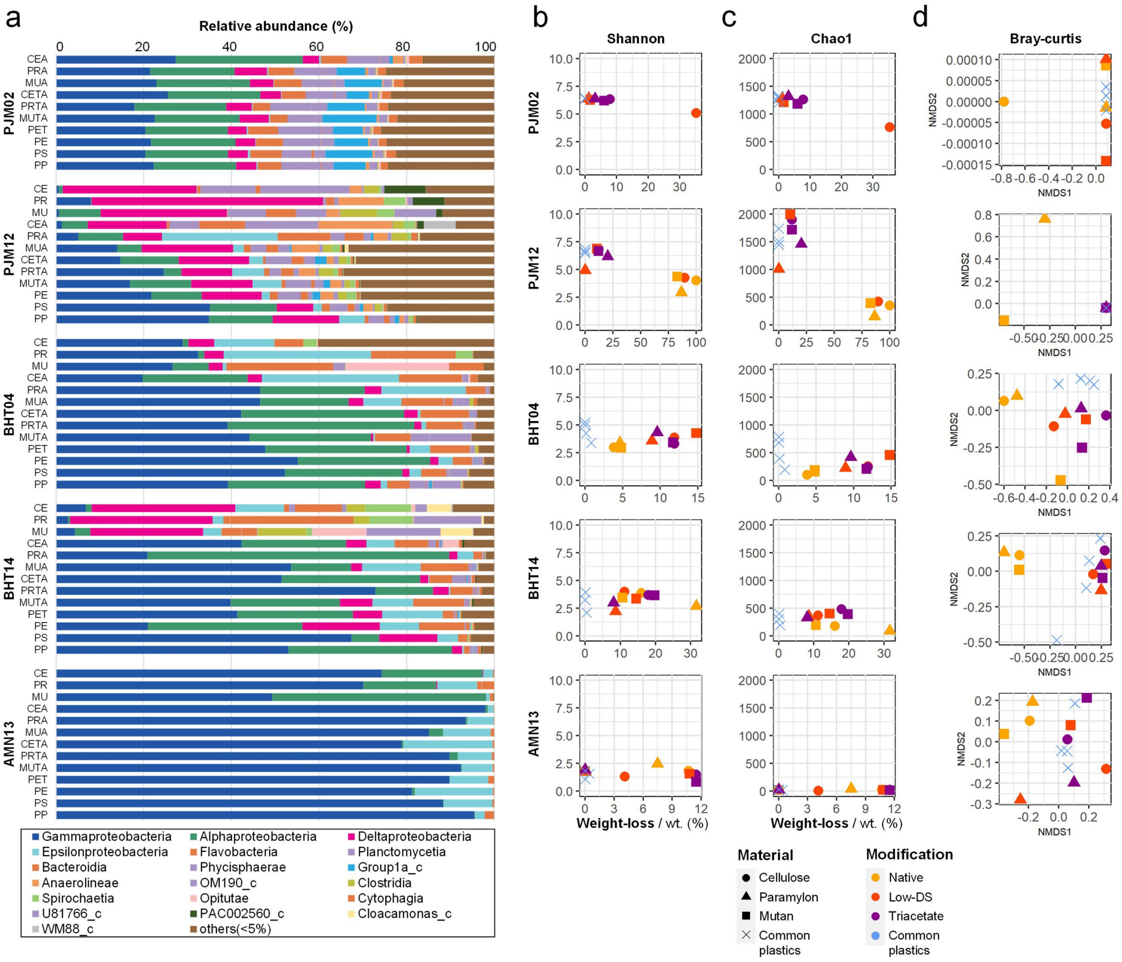

Supplementary Fig. 18 | Polysaccharide-associated bacterial communities vary by degree of acetylation, site, and immersion period.

The data for three native samples at PJM02 could not be obtained because of sample degradation. **a,** 16S rRNA gene-based community compositions at the class level in biofilms on polysaccharides. “Others” are classes representing <5% relative abundance. **b** and **c**, Correlation between material degradation and diversity of prokaryotic communities of each material shown in terms of the Shannon index **b** and Chao1 index **c**. **d**, Non-metric multidimensional scaling (NMDS) ordination of bacterial community composition between polysaccharides and common plastics using Bray–Curtis dissimilarity. NMDS stress value: 0.000084 (PJM02), 0.000085 (PJM12), 0.1099 (BHT04), 0.064523 (BHT14), and 0.113100 (AMN13). These plots show similarity between the communities on acetylated polysaccharides and those on common plastics.

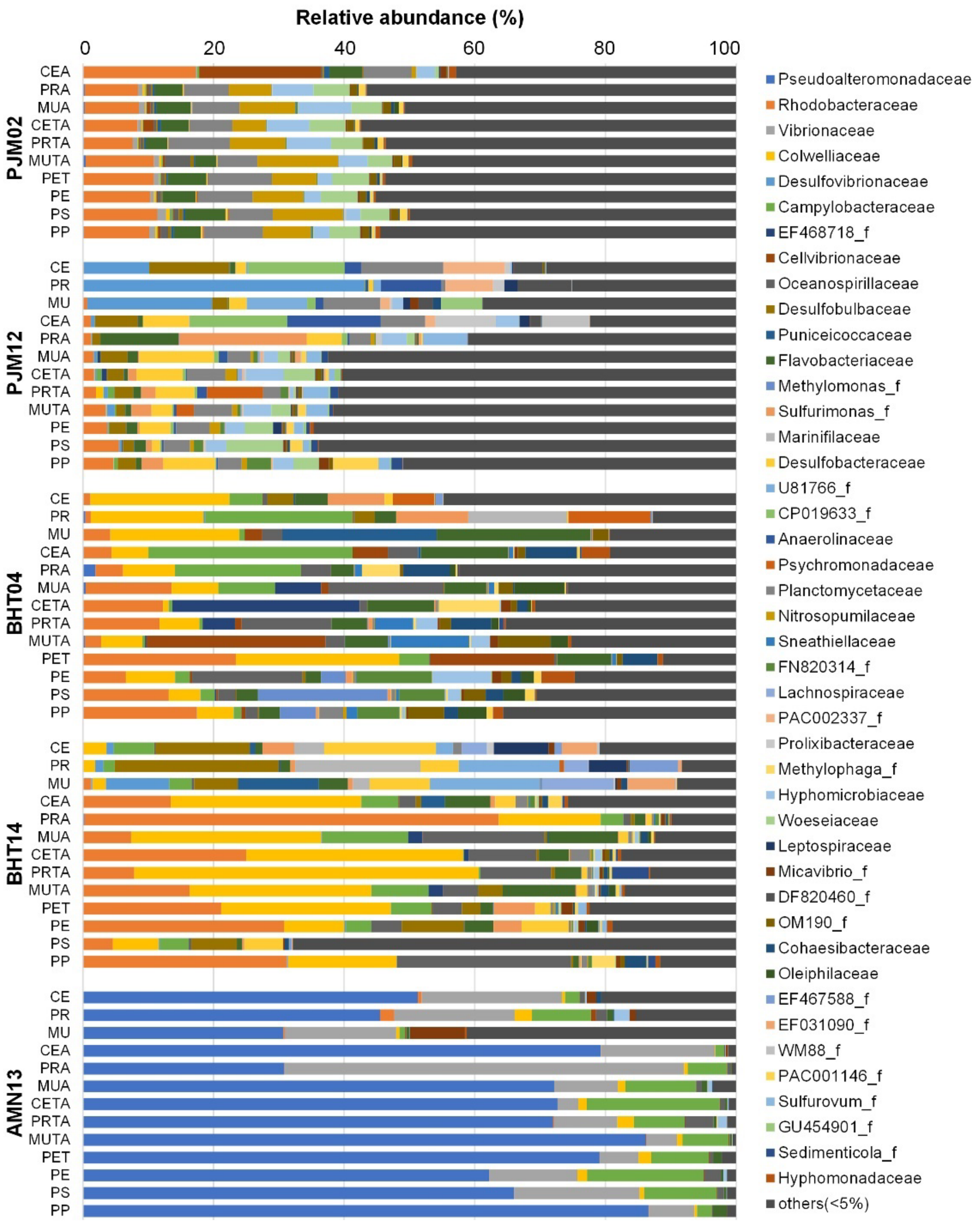

Supplementary Fig. 19 | 16S rRNA gene-based prokaryotic community composition of polysaccharides, polysaccharide ester derivatives and common plastics at the family level. "Others" are families representing <5% relative abundance; the data for three native samples at PJM02 could not be obtained because of sample degradation.

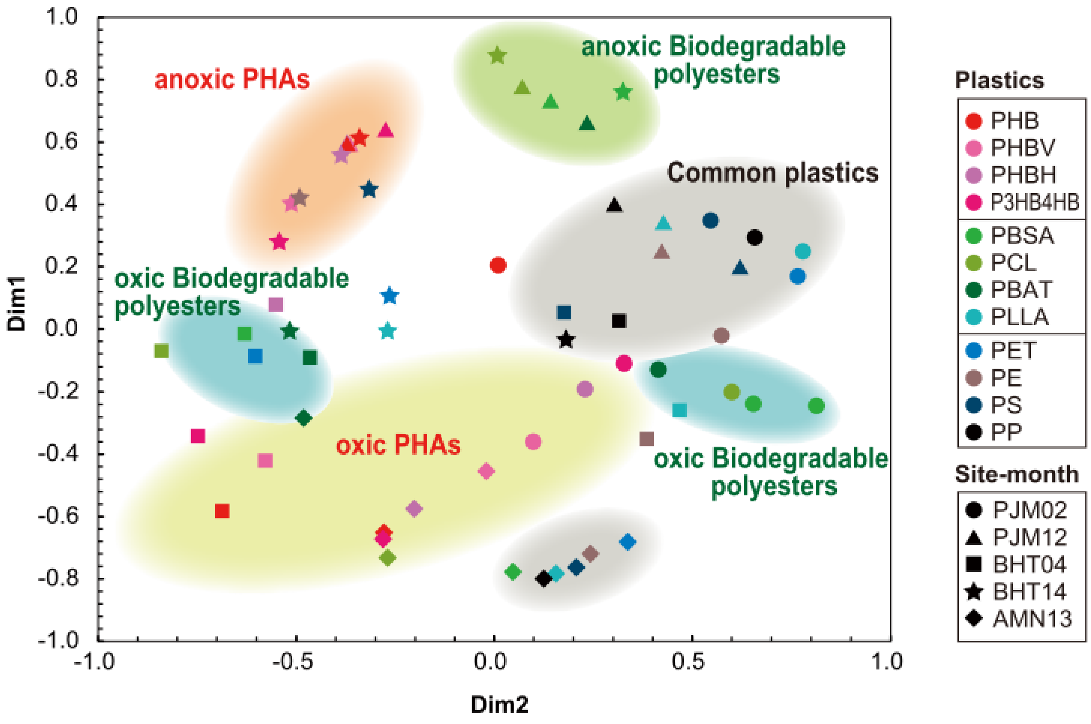

Supplementary Fig. 20 | Comparison of microbial community compositions of the plastispheres based on a multidimensional scale plot.

Multidimensional scale (MDS) plot is analyzed based on microbial community composition described in Supplementary Fig. 14 with sub-division of *Gammaproteobacteria*, *Alphaproteobacteria*, and *Deltaproteobacteria*.

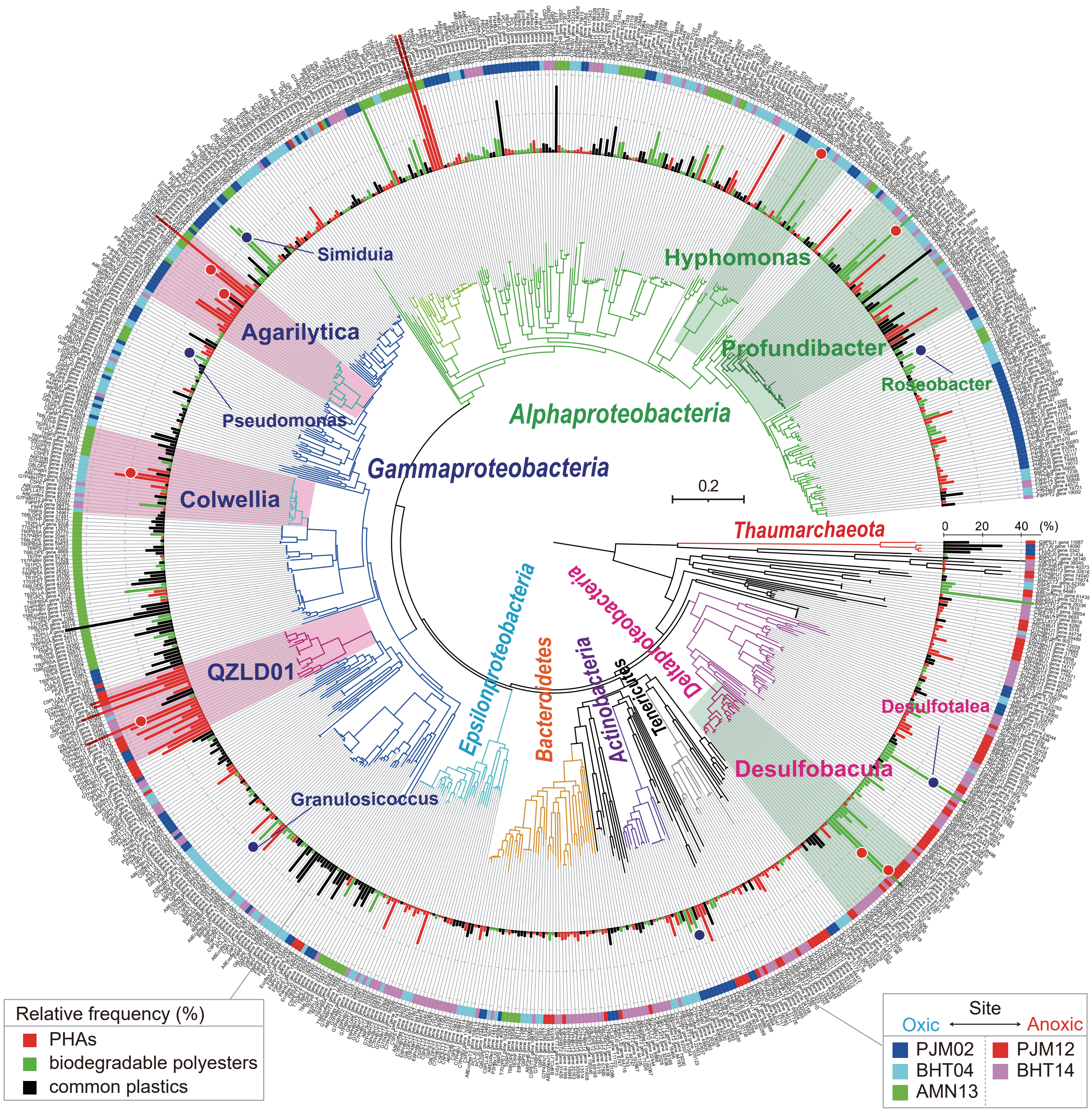

Supplementary Fig. 21 | Phylogenetic tree of single-copy housekeeping gene, ribosomal protein S3, RpsC, identified in plastisphere metagenomes at the sea.

Phylogenetic tree of ribosomal protein S3 (RpsC) identified in all metagenomic assemblies is shown. Taxonomy of the proteins is shown by different color of nodes. Relative frequency within the communities is shown by bar chart outside the tree with different colors (red, PHAs; green, biodegradable polyesters; black, common plastics). The site and immersion time are shown as color strip outside of the bar chart. Dominant MAGs used for subsequent analysis are marked by a red dot, while their taxonomies are also described. Another six MAGs show in biodegradable polyesters are marked by a blue dot. Gene ID coding the RpsC protein is described outside of the color strip.

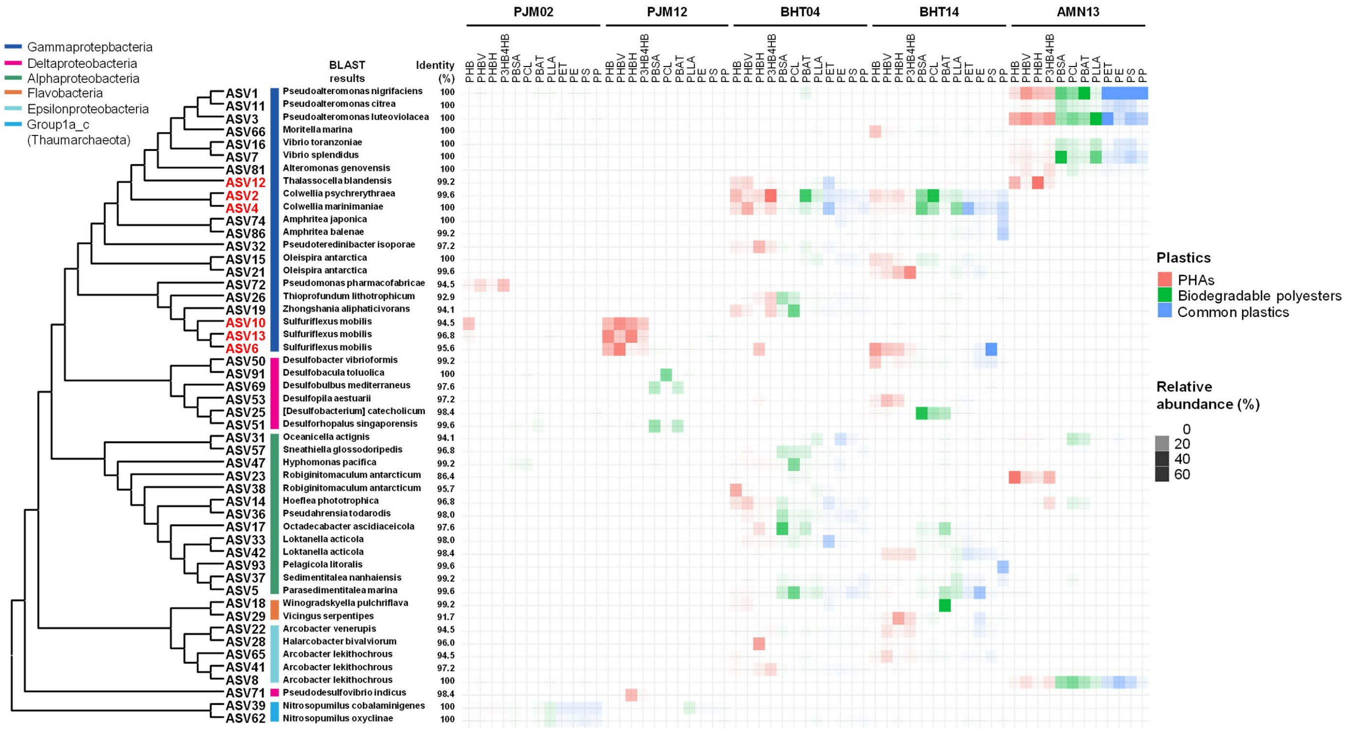

Supplementary Fig. 22 | Shade plot illustrating the relative abundances (%) of plastic-specific amplicon sequence variants (ASVs) at different sites and periods of immersion.

The top 50 most abundant ASVs (assigned by specific numbers) across the gross relative abundance in all plastispheres on 12 plastics in five samples at three marine sites are indicated. Relative abundances of ASVs among different plastics, sites, and periods of immersion are displayed by shading of cells—darker represents higher abundance. Blocks are colored to indicate the classification of plastics: polyhydroxyalkanoate (PHA) plastics marked as red include PHB, PHBV, PHBH, and P3HB4HB; biodegradable polyesters marked as green include PLLA, PBSA, PCL, and PBAT; common plastics marked as blue include PE, PP, PS, and PET. The horizontal axis shows the type of plastic associated with the plastisphere in each sampling. On the vertical axis, a phylogenetic tree shows the top 50 ASVs. Classification at the class level is indicated by color. The closest taxonomic species identified by BLAST search and the identity (%) are also shown.

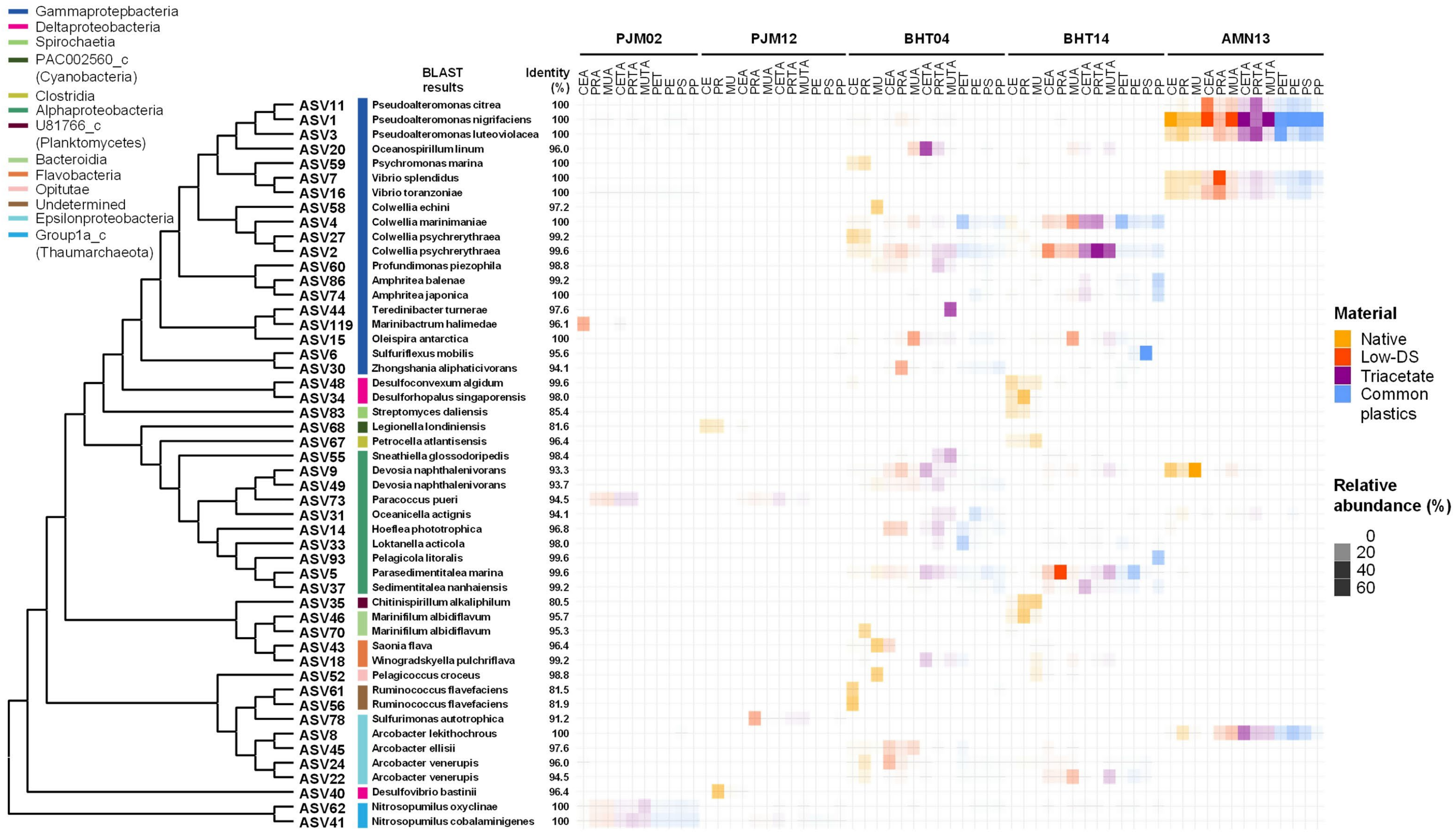

Supplementary Fig. 23 | Shade plot of the 50 most abundant ASVs in biofilms obtained from all polysaccharides and non-biodegradable common plastics. A phylogenetic tree of ASVs was constructed using the neighbor-joining method. Color bars on the right-side of ASV numbers show the class to which the ASV belongs. The heatmap shows the distribution of dominant ASVs (>10% total reads) in anaerobic enrichment cultures from Atlantis Massif subseafloor rocks. The ASV name, its class affiliation, the name of its closest species, and 16S rRNA gene percentage identity with this closest homolog are indicated on the right.

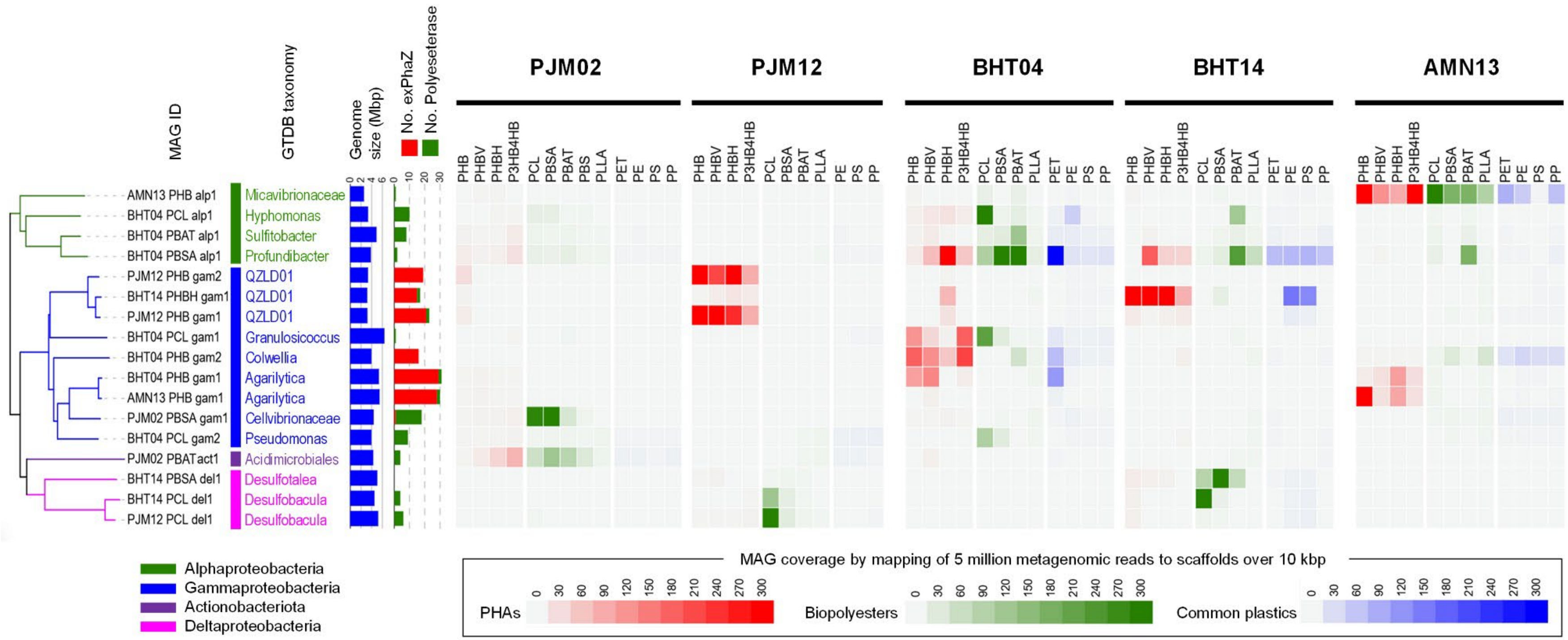

Supplementary Fig. 24 | Shade plot illustrating the relative abundances of plastic specific metagenome-assembled genomes (MAGs) at different sites and periods of immersion.

The 17 highly abundant MAGs across the gross relative abundance of all plastispheres on twelve plastics in five sampling at three marine sites were indicated. Relative abundances of MAGs among different plastics, sites, and periods of immersion were displayed by shading of cells, darker represented higher abundance. Cells were colored to indicate the classification of plastics. Polyhydroxyalkanoate (PHA) plastics marked as red including PHB, PHBV, PHBH, and P3HB4HB; biopolyester plastics marked as green including PLLA, PBSA, PBS, PCL, and PBAT; common plastics marked as blue including PE, PP, PS, and PET. The horizontal axis showed types of plastics to obtain plastispheres in each sampling. The vertical axis showed a phylogenomic tree of the 17 MAGs along with GTDB taxonomy, genome size, and number of degrading enzymes (secreted PHB depolymerase, exPhaZ, and polyesterase) coded in the MAGs.

a

Site_MAG ID	PpsC-based taxonomy	Oxic/ Anoxic	OTU	Relative frequency	Genome Size (bp)	No. Scaffolds	PHB depolymerases	Cuttinases/ Polyesterases
**BHT04**_PCL gam1	Granulosicoccus	Oxic	ASV019	14.2%	6,319,541	642	0	1
**BHT04**_PCL gam2	Pseudomonas	Oxic	ASV095	6.8%	3,889,687	23	0	**9**
**PJM02**_PBAT act1	Actinobacteria	Oxic	ASV142	10.7%	4,238,728	296	0	**4**
**BHT04**_PBAT alp2	others (Alpha)	Oxic	ASV065	7.8%	4,833,856	61	0	**8**
**PJM02**_PBSA gam1	Simiduia	Oxic	ASV141	32.1%	4,307,390	14	1	**17**
**BHT14**_PBSA del1	Desulfotalea	Anoxic	ASV025	49.9%	4,989,220	165	0	0

b

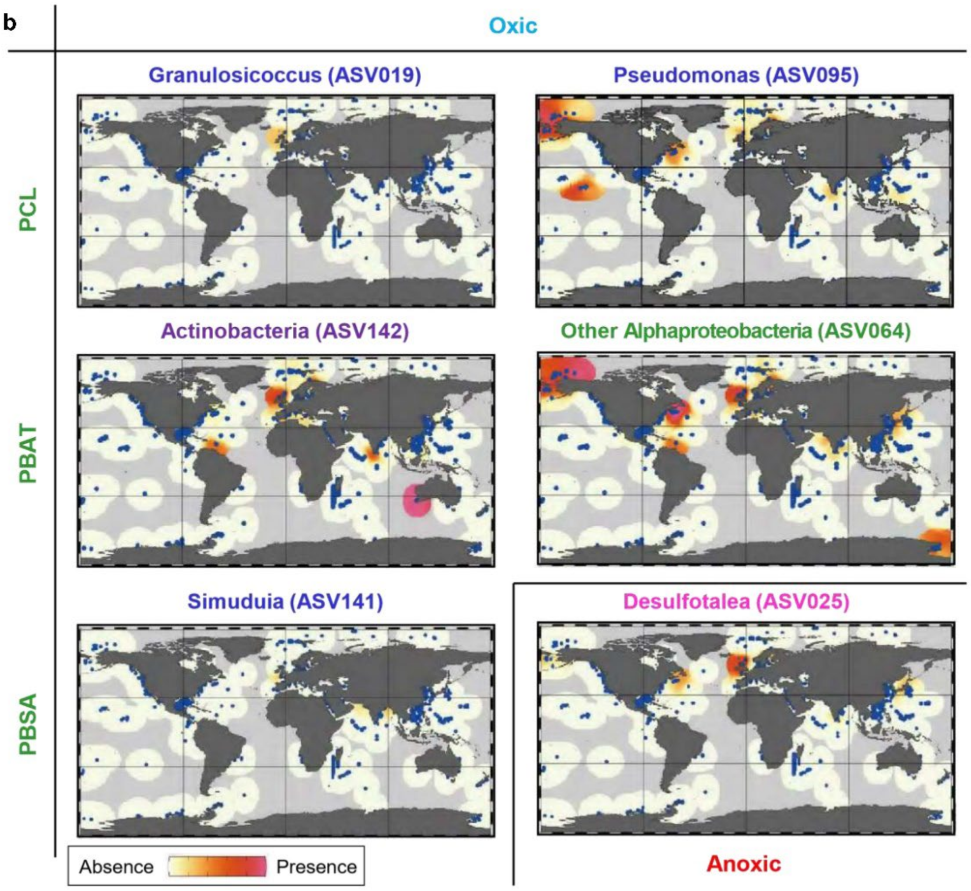

Supplementary Fig. 25 | Additional information for MAGs of dominant microbes within plastisphere established on biodegradable polyesters at the sea.

a, Stats of metagenome-assembled genomes (MAGs) abundantly observed within the plastispheres of biodegradable polyesters under oxic/anoxic conditions. Number of key genes related to biodegradation of the plastics are described for each MAG. **b,** The global distribution of the six potential degraders of biodegradable polyesters (PCL, PBAT, or PBSA) in the marine sediment. Full length 16S rRNA sequences from each MAG were used for the analysis with the reference of publicly available 16S rRNA surveys.

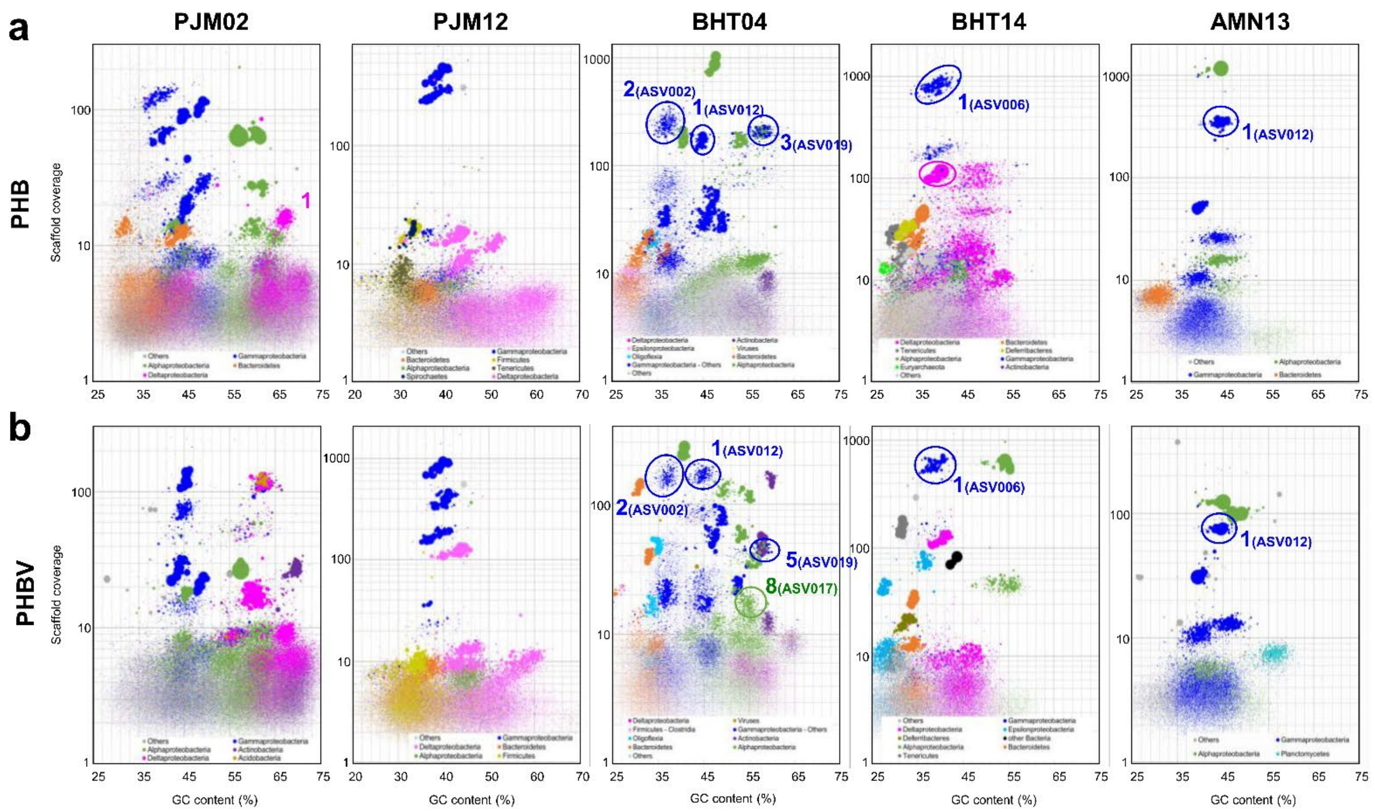

Supplementary Fig. 26 | Genome clustering from metagenome sequences of plastisphere for a, PHB and b, PHBV established under oceanic condition.

Genome clusters from the assembled scaffolds from metagenomic sequences of plastisphere under different oceanic location, water depth, and time were identified using the estimated taxonomic classification (color of dots), length (size of dots), GC content (X-axis) and mean coverage of scaffold (Y-axis). Colored circles on PHB plastispheres (panel a) indicate clusters of individual metagenome-assembled genomes (MAGs), while numbers shown near the circles correspond to the MAG IDs established from plastisphere PHBs.

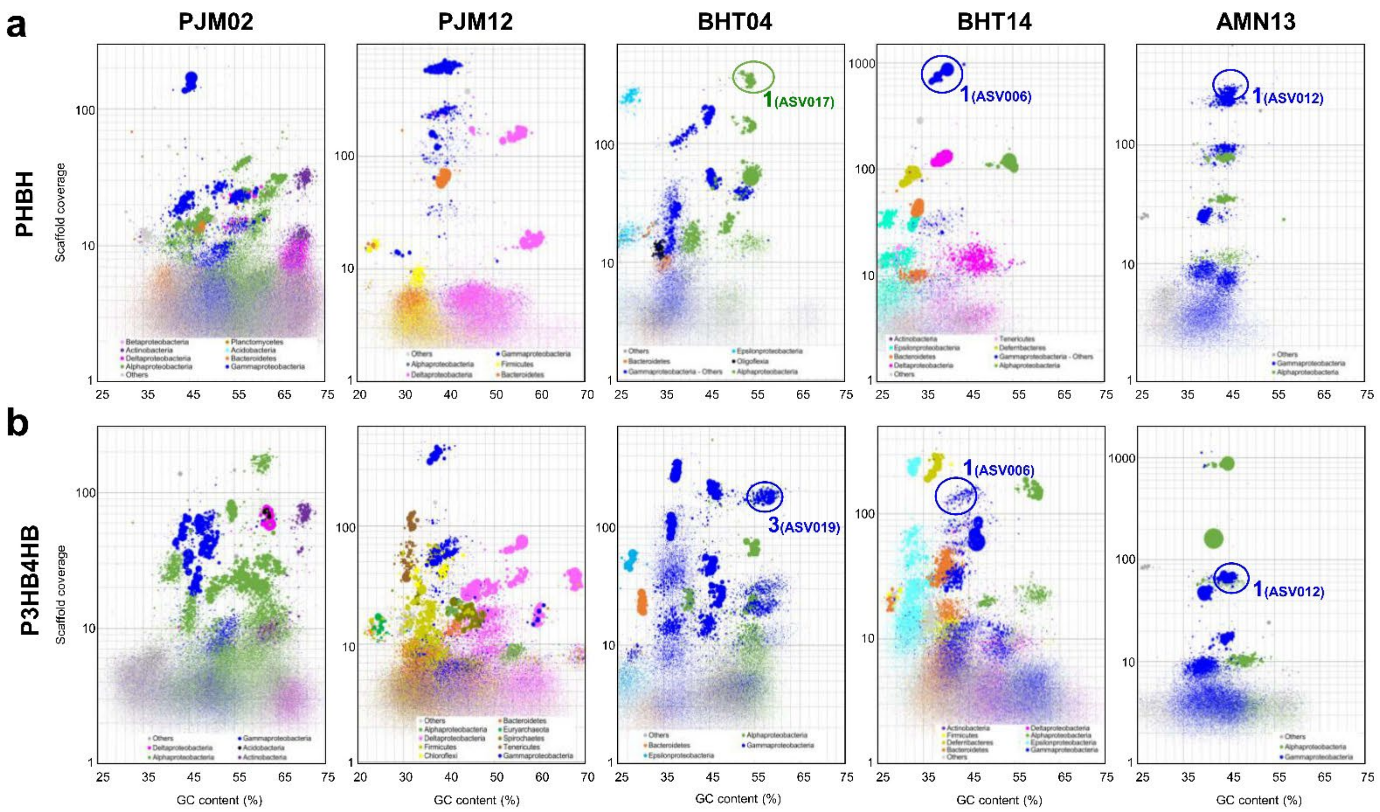

Supplementary Fig. 27 | Genome clustering from metagenome sequences of plastisphere for a, PHBH and b, P3HB4HB established under oceanic condition.

Genome clusters from the assembled scaffolds from metagenomic sequences of plastisphere under different oceanic location, water depth, and time were identified using the estimated taxonomic classification (color of dots), length (size of dots), GC content (X-axis) and mean coverage of scaffold (Y-axis).

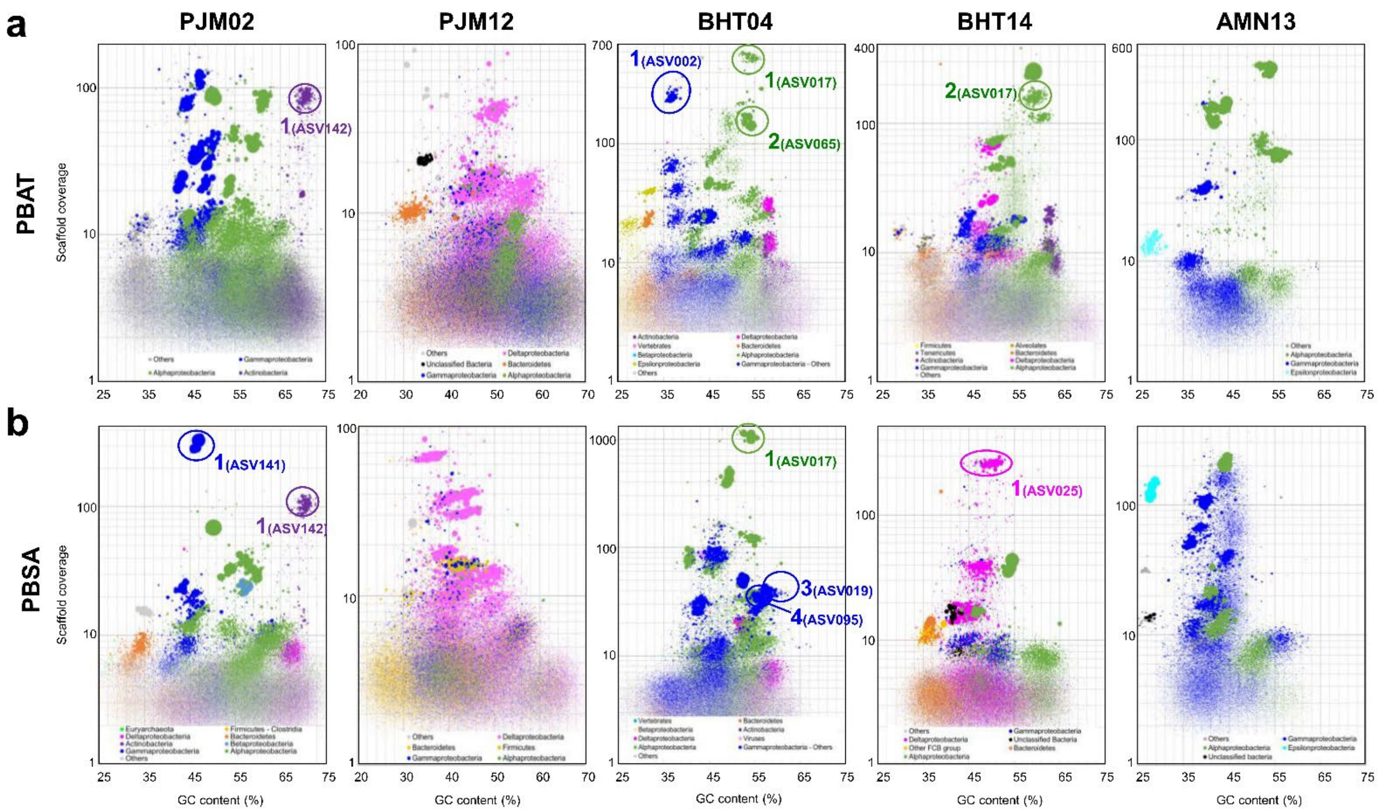

Supplementary Fig. 28 | Genome clustering from metagenome sequences of plastisphere for a, PBAT and b, PBSA established under oceanic condition.

Genome clusters from the assembled scaffolds from metagenomic sequences of plastisphere under different oceanic location, water depth, and time were identified using the estimated taxonomic classification (color of dots), length (size of dots), GC content (X-axis) and mean coverage of scaffold (Y-axis).

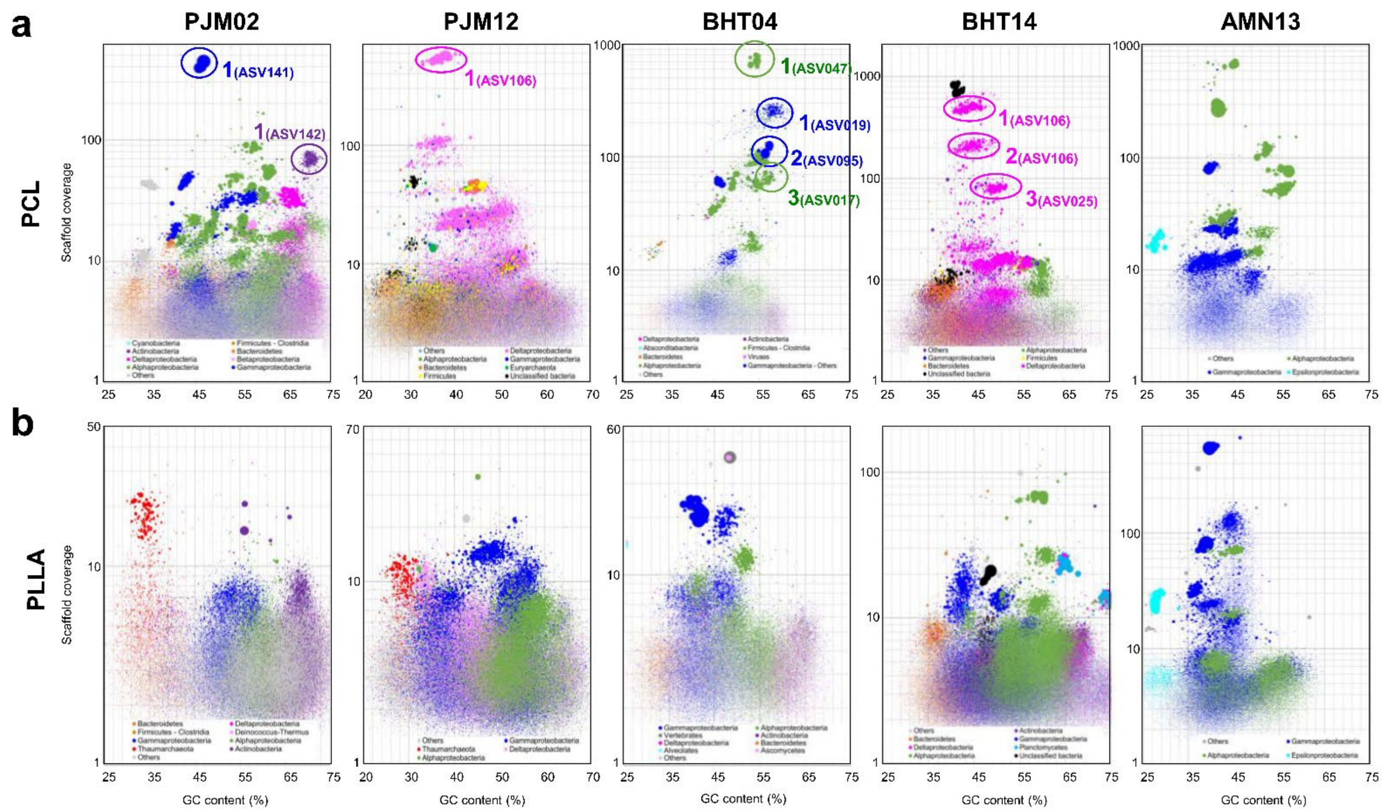

Supplementary Fig. 29 | Genome clustering from metagenome sequences of plastisphere for a, PCL and b, PLLA established under oceanic condition.

Genome clusters from the assembled scaffolds from metagenomic sequences of plastisphere under different oceanic location, water depth, and time were identified using the estimated taxonomic classification (color of dots), length (size of dots), GC content (X-axis) and mean coverage of scaffold (Y-axis).

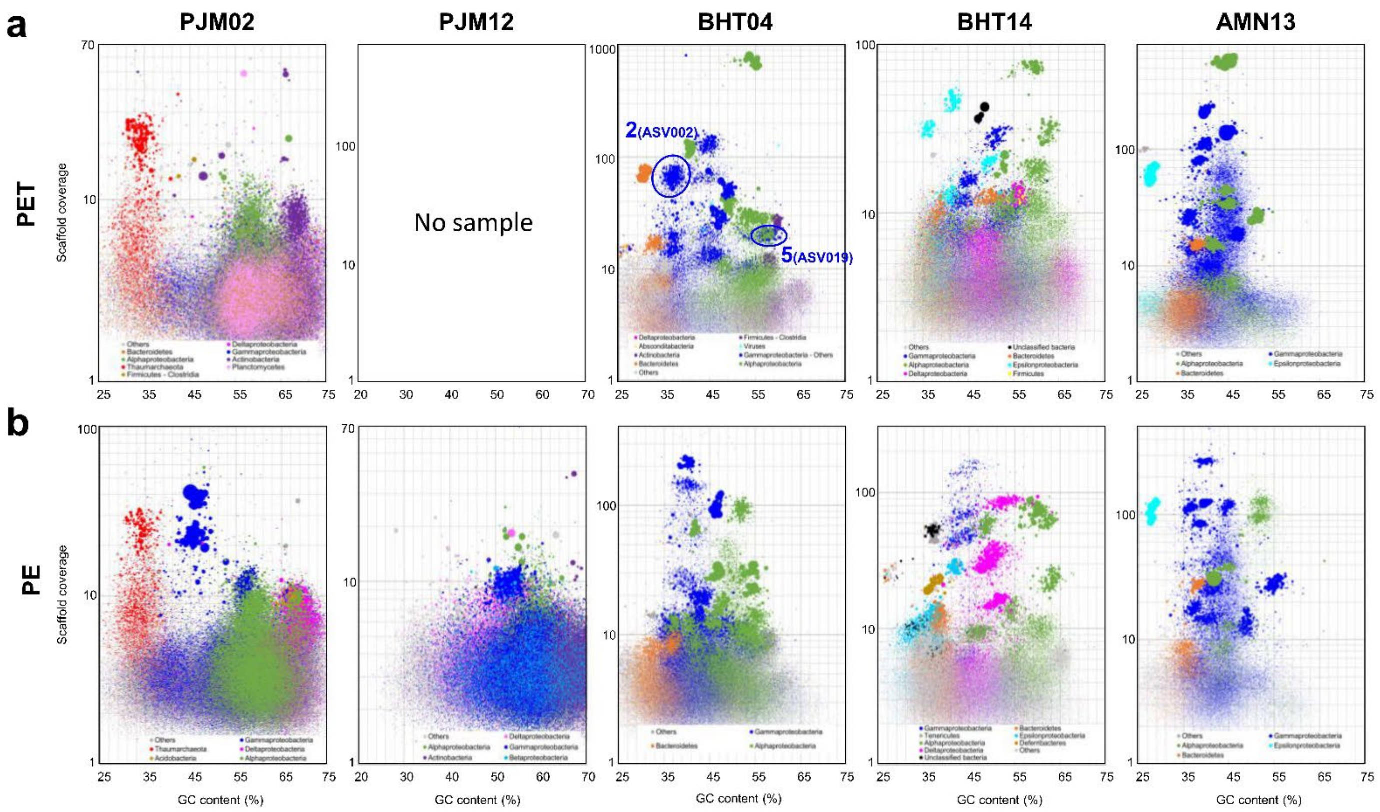

Supplementary Fig. 30 | Genome clustering from metagenome sequences of plastisphere for a, PET and b, PE established under oceanic condition.

Genome clusters from the assembled scaffolds from metagenomic sequences of plastisphere under different oceanic location, water depth, and time were identified using the estimated taxonomic classification (color of dots), length (size of dots), GC content (X-axis) and mean coverage of scaffold (Y-axis).

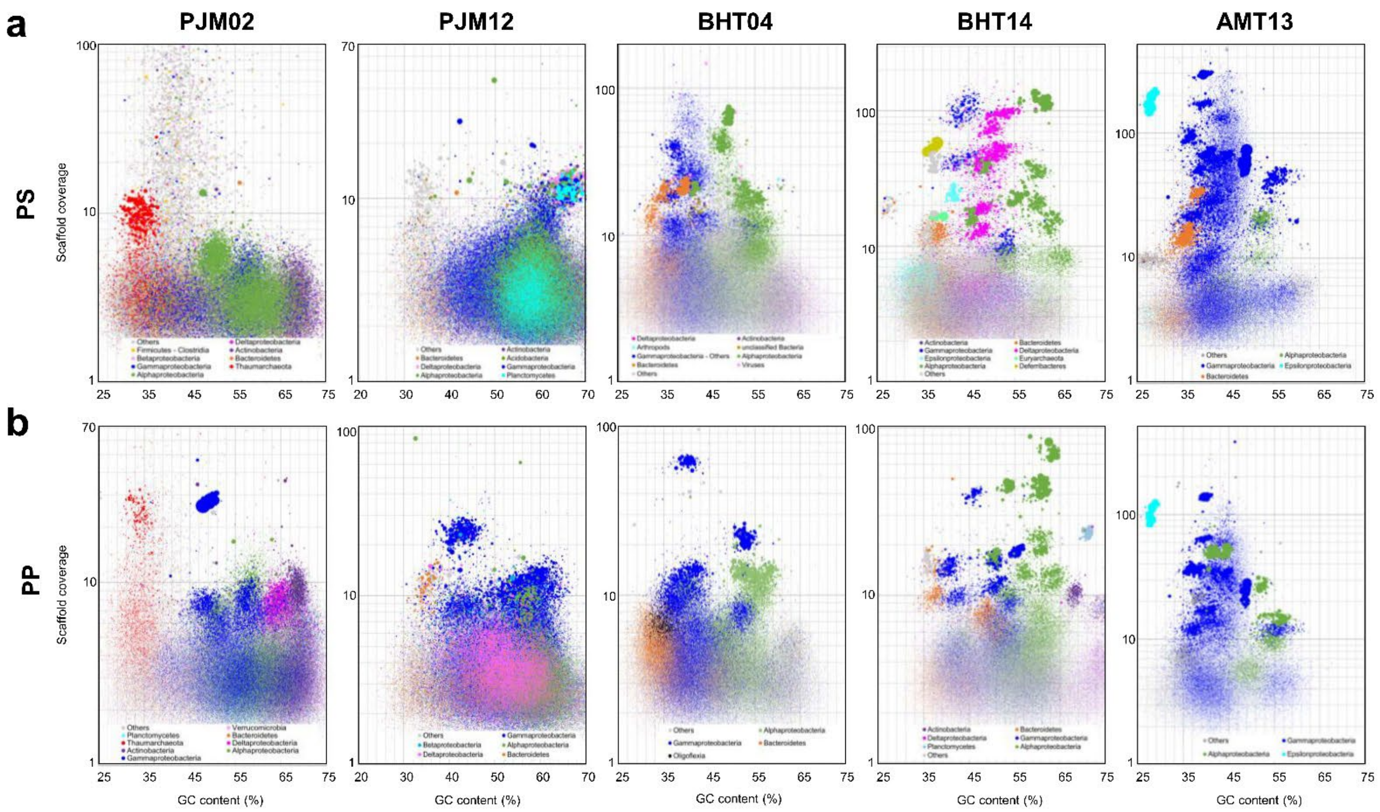

Supplementary Fig. 31 | Genome clustering from metagenome sequences of plastisphere for a, PS and b, PP established under oceanic condition.

Genome clusters from the assembled scaffolds from metagenomic sequences of plastisphere under different oceanic location, water depth, and time were identified using the estimated taxonomic classification (color of dots), length (size of dots), GC content (X-axis) and mean coverage of scaffold (Y-axis).

Supplementary Table 1 | Detail of samples placed for 3 months at BHT (BHT03).

Sample	Weight decrease / mg	Weight loss / wt%	Thickness $_{(before)}$ / μm	Thickness $_{(after)}$ / μm	Biodegradation rate / $\mu g \cdot cm^{-2} \cdot day^{-1}$
PHB	60.9±6.8	10.9±1.6	308.2±17.7	275.7±7.3	19.8±2.3
PHBV	65.4±19.8	16.8±5.5	216.4±13.5	200.6±5.0	22.0±6.6
PHBH	86.9±19.8	21.6±6.5	221.6±20.7	189.9±24.6	28.6±6.7
P3HB4HB	140.0±35.3	26.3±6.6	290.1±14.8	258.5±2.7	45.8±11.5
PCL	8.9±1.0	1.9±0.1	262.7±39.9	266.3±38.0	2.9±0.3
PBS	0.0±5.0	0.0±0.9	266.4±22.9	278.9±18.8	0.0±0.1
PBSA	10.3±1.4	1.8±0.0	291.4±0.0	291.1±0.0	3.4±0.0
PBAT	6.8±0.3	1.5±0.0	240.4±21.6	243.4±11.2	2.2±0.1
PLLA	-0.3±0.7	0.0±0.1	273.9±23.8	280.8±18.5	-0.1±0.2
PP	0.1±0.2	0.0±0.1	219.9±21.7	220.7±17.7	0.0±0.1
PE	0.2±0.1	0.1±0.0	268.5±4.7	260.6±-1.4	0.1±0.0
PS	-0.4±0.4	-0.1±0.1	110.5±0.1	102.7±-0.3	-0.1±0.1
PET	0.2±0.1	0.1±0.0	240.9±16.5	232.9±15.7	0.1±0.0

Supplementary Table 2 | Detail of samples placed for 8 months at BHT (BHT08).

Sample	Weight decrease / mg	Weight loss / wt%	Thickness (before) / µm	Thickness (after) / µm	Biodegradation rate / $\mu g \cdot cm^{-2} \cdot day^{-1}$
PHB	195.5±19.3	35.5±3.6	287.3±11.8	226.6±14.4	24.4±2.4
PHBV	213.2±25.4	46.3±9.3	253.1±22.8	170.8±34.6	27.1±3.2
PHBH	219.8±44.8	51.8±11.1	229.0±21.9	154.6±29.6	27.5±5.6
P3HB4HB	529.1±60.1	90.8±11.1	313.6±29.1	102.8±84.4	65.1±7.8
PCL	10.8±1.5	2.3±0.2	289.8±25.9	266.8±19.5	1.3±0.2
PBS	3.4±0.4	0.7±0.1	267.1±18.4	260.6±20.2	0.4±0.0
PBSA	12.0±0.9	2.2±0.0	307.4±0.0	281.8±0.0	1.5±0.0
PBAT	8.0±0.5	1.7±0.1	252.0±24.1	241.6±26.3	1.0±0.1
PLLA	0.3±0.2	0.1±0.0	285.3±13.5	281.1±16.2	0.0±0.0
PP	0.2±0.0	0.1±0.0	214.7±20.5	211.3±28.5	0.0±0.0
PE	0.3±0.3	0.1±0.1	258.8±40.0	241.6±29.4	0.0±0.0
PS	0.2±0.1	0.0±0.0	277.0±32.6	246.1±36.9	0.0±0.0
PET	0.4±0.3	0.2±0.1	104.4±0.0	95.8±0.4	0.0±0.0

Supplementary Table 3 | Biodegradation rate of injection-molded plastic samples.

Place	Biodegradation rate / $\mu g \cdot cm^{-2} \cdot day^{-1}$												
	PHB	PHBV	PHBH	P3HB4HB	PBS	PBSA	PCL	PBAT	PLLA	PP	LDPE	PET	PS
PJM12	191.0	145.1	188.2	173.0	7.4	41.2	51.6	22.3	-0.7	0.0	0.4	0.2	-0.1
BHT04	3.2	6.4	5.3	28.1	-2.1	7.3	7.0	16.1	-1.5	0.2	0.9	-58.2	1.2
BHT14	19.2	20.3	23.6	35.3	-0.3	1.8	2.9	5.1	-0.2	0.0	0.3	0.2	0.4
AMN13	5.2	2.3	2.1	5.8	-1.4	0.1	1.4	2.3	-1.3	0.0	0.0	0.1	0.0

Supplementary Table 4 | Biodegradation rates of various melt-pressed PHA and biodegradable polyester films.

Place	Biodegradation rate / $\mu g \cdot cm^{-2} \cdot day^{-1}$								
	PHB	PHBV	PHBH	P3HB4HB	PBS	PBSA	PCL	PBAT	PLLA
PJM02	106.6±29.3	122.5±19.7	121.3±27.8	147.7±17.6	7.8±3.2	91.0±8.4	118.1±9.8	49.1±15.9	0.4±0.1
BHT03	19.8±2.3	22.0±6.6	28.6±6.7	45.8±11.5	0.0±0.1	3.4±0.4	2.9±0.3	2.2±0.1	-0.1±0.2
BHT08	24.4±2.4	27.1±3.2	27.5±5.6	65.1±7.8	0.4±0.0	1.5±0.0	1.3±0.2	1.0±0.1	0.0±0.0
BMS05	12.8±2.3	17.6±1.6	13.3±2.1	31.3±6.0	2.2±0.1	2.4±0.3	2.9±0.4	2.3±0.2	0.0±0.1
BMJ05	10.9±1.1	n.d.	n.d.	n.d.	0.1±0.0	0.4±0.1	2.5±0.4	0.3±0.1	0.1±0.1
AKR05	5.5±2.0	n.d.	n.d.	n.d.	0.3±0.2	0.3±0.1	0.8±0.0	0.3±0.2	0.1±0.1
AMN13	4.7	4.9	3.2	4.4	0.1	1.8	1.0	2.3	0.7

Supplementary Table 5 | Detail of samples placed for 2 months at PJM (PJM02).

Sample	Weight decrease / mg	Weight loss / wt%	Thickness $_{(before)}$ / µm	Thickness $_{(after)}$ / µm	Biodegradation rate / $\mu g \cdot cm^{-2} \cdot day^{-1}$
PHB	208.7±58.3	30.7±6.2	342.1±44.6	303.8±37.9	106.6±29.3
PHBV	243.7±37.1	42.6±8.2	308.8±21.9	228.4±21.4	122.5±19.7
PHBH	238.6±53.3	39.9±11.6	324.8±27.4	247.4±43.8	121.3±27.8
P3HB4HB	292.1±34.0	39.1±5.2	395.3±40.5	301.0±42.0	147.7±17.6
PCL	233.1±20.1	34.0±4.5	385.6±50.6	306.7±38.3	118.1±9.8
PBS	15.6±6.5	2.7±1.0	297.2±21.6	296.5±16.2	7.8±3.2
PBSA	181.2±14.4	31.2±4.5	314.8±27.3	254.9±32.5	91.0±8.4
PBAT	97.9±31.1	12.4±3.9	383.8±20.3	370.3±20.4	49.1±15.9
PLLA	0.8±0.2	0.1±0.0	353.7±19.6	345.5±20.9	0.4±0.1
PP	-1.3±0.5	-0.2±0.1	391.2±46.2	424.8±25.2	-0.7±0.3
PE	-0.1±1.2	0.0±0.2	493.8±60.2	486.4±71.2	0.0±0.6
PS	-0.8±0.4	0.0±0.1	404.9±32.1	409.8±34.2	-0.4±0.2
PET	-0.1±0.6	-0.1±0.3	112.3±3.1	106.2±2.4	-0.1±0.3

Supplementary Table 6 | Detail of samples placed for 5 months at BMS (BMS05).

Sample	Weight decrease / mg	Weight loss / wt%	Thickness $_{(before)}$ / µm	Thickness $_{(after)}$ / µm	Biodegradation rate / µg·cm^{-2}·day^{-1}
PHB	56.1±10.5	10.7±3.0	346.8±34.7	272.1±38.7	12.8±2.3
PHBV	76.3±8.0	15.1±3.1	304.6±44.4	256.0±42.0	17.6±1.6
PHBH	58.8±9.1	9.1±1.0	333.0±26.8	317.9±24.3	13.3±2.1
P3HB4HB	136.1±25.7	20.1±3.8	364.7±22.5	318.4±21.5	31.3±6.0
PCL	12.7±1.6	1.6±0.1	437.2±32.4	443.4±29.9	2.9±0.4
PBS	9.9±0.3	2.0±0.3	261.2±22.9	252.5±22.7	2.2±0.1
PBSA	10.5±1.4	1.6±0.1	349.5±43.0	330.2±35.0	2.4±0.3
PBAT	10.2±1.0	1.3±0.1	394.4±52.2	399.2±56.6	2.3±0.2
PLLA	-0.1±0.4	0.0±0.1	317.6±69.7	304.0±26.6	0.0±0.1
PP	-0.1±0.2	0.0±0.0	469.4±54.8	375.8±47.4	0.0±0.0
PE	0.0±0.1	0.0±0.0	276.8±14.2	257.5±8.3	0.0±0.0
PS	-1.2±0.1	-0.2±0.0	376.1±54.7	362.9±44.5	-0.3±0.0
PET	0.0±0.0	0.0±0.0	140.2±12.8	98.9±0.9	0.0±0.0

Supplementary Table 7 | The weight-loss of polysaccharide esters derivatives films.

Place	Weight-loss / wt%								
	CE	CEA	CETA	PR	PRA	PRTA	MU	MUA	MUTA
PJM02	100.0	89.6	12.1	86.4	-1.6	20.3	82.8	10.5	12.0
BHT14	16.0	11.2	17.8	31.6	8.6	27.6	10.6	14.5	19.7
AMN13	10.7	4.1	11.5	7.5	-1.1	-0.8	-2.2	10.8	11.5

Supplementary Table 8 | Stats for metagenomic assemblies of plastisphere at the sea.

Sites		Plastics	No. of sequence reads	No. of mapped reads to scaffolds	Total bases (bp)	No. Scaffolds	No. ORFs	Opt. Temp. (°C)	Zc	Community header
PJM02	PHAs	PHB	63,657,156	30,009,225	278,378,781	229,659	375,977	25.8	-0.142	P3HBx1J0
		PHBV	42,053,848	26,821,635	236,142,568	165,658	329,451	33.0	-0.125	PHBVJ0
		PHBH	59,318,544	28,760,180	387,517,549	307,419	572,164	28.8	-0.135	PHBHJ0
		P3HB4HB	49,119,126	38,601,663	265,344,151	150,104	358,120	27.1	-0.134	P4HBJ0
	biodegradable polyesters	PBAT	57,343,266	34,737,377	310,571,323	217,966	451,751	27.6	-0.139	EcoflexJ0
		PBSA	46,786,658	29,909,958	254,983,246	171,295	369,354	28.8	-0.139	PBSAJ0
		PBS	61,568,798	20,168,669	260,465,820	235,482	410,602	28.8	-0.127	PBSJ0
		PCL	72,922,830	50,189,626	415,487,352	274,798	584,398	28.6	-0.137	PCLJ0
		PLLA	49,813,652	4,006,855	101,007,345	133,648	190,384	33.0	-0.128	PLLAJ0
	common	PET	35,082,442	2,967,428	132,749,920	183,385	249,466	35.4	-0.131	PETJ0
		PE	56,323,142	8,855,751	215,003,864	253,909	369,428	36.2	-0.134	LDPEJ0
		PS	35,676,402	5,779,442	95,187,166	137,881	101,395	32.8	-0.140	PSJ0
		PP	69,876,690	9,484,315	207,208,658	246,290	352,336	33.4	-0.133	PPJ0
PJM12	PHAs	PHB	23,266,982	19,654,896	92,771,385	69,489	135,640	29.5	-0.164	A6P3HBJ1
		PHBV	45,922,320	41,309,739	109,881,412	71,843	152,731	30.3	-0.165	C7PHBVJ1
		PHBH	50,126,496	45,439,951	82,229,992	54,569	119,945	30.8	-0.162	E6PHBHJ1
		P3HB4HB	47,017,780	40,741,922	266,734,324	123,709	334,218	36.6	-0.171	G7P4BHJ1
	biodegradable polyesters	PBAT	52,133,952	13,948,188	260,742,308	242,190	411,886	33.6	-0.166	A8EcoflexJ1
		PBSA	37,322,874	18,355,420	224,885,468	164,445	316,331	31.2	-0.172	B8PBSAJ1
		PCL	55,680,592	41,040,080	253,163,885	179,210	352,422	28.7	-0.179	B9PCLJ1
		PLLA	56,065,394	5,254,785	154,285,706	194,132	284,779	34.9	-0.148	C9PLLAJ1
	common	PE	45,448,040	3,969,254	134,548,675	174,260	253,278	33.8	-0.139	G8LDPEJ1
		PS	63,535,638	4,629,370	148,014,386	196,718	283,065	39.6	-0.134	G9PSJ1
		PP	44,075,486	6,211,090	163,025,025	181,201	285,031	33.9	-0.146	F9PPJ1
BHT04	PHAs	PHB	82,147,782	74,923,722	182,494,978	104,721	247,090	15.8	-0.149	A6P3HB
		PHBV	64,812,168	58,579,753	232,290,094	104,226	282,518	22.9	-0.153	C7PHBV
		PHBH	47,604,546	42,784,603	132,803,468	61,576	167,402	24.1	-0.161	E6PHBH
		P3HB4HB	54,555,588	49,458,423	149,841,116	78,539	199,784	24.1	-0.157	G7P4BH
	biodegradable polyesters	PBAT	77,449,734	67,551,185	305,344,113	171,670	399,932	21.8	-0.153	A8Ecoflex
		PBSA	81,400,242	72,179,437	216,488,780	120,954	282,394	22.1	-0.148	B8PBSA
		PCL	58,436,592	52,879,819	126,574,727	72,778	171,274	24.1	-0.141	B9PCL
		PLLA	12,600,822	4,167,724	77,910,307	76,487	124,685	25.4	-0.153	C9PLLA
	common	PET	59,591,110	50,783,177	207,664,125	124,706	275,794	21.7	-0.153	C5PET
		PE	44,292,228	28,859,392	259,753,657	188,352	378,121	24.4	-0.153	G8LDPE
		PS	49,164,004	25,719,217	304,294,407	246,958	467,798	25.6	-0.157	G9PS
		PP	29,810,292	10,971,980	208,597,656	195,491	331,074	24.0	-0.152	F9PP
BHT14	PHAs	PHB	51,000,188	44,365,457	194,259,820	99,601	243,152	28.6	-0.174	A6P3HBT2
		PHBV	54,569,286	49,078,825	103,244,766	51,927	132,028	27.3	-0.167	C7PHBVT2
		PHBH	39,961,876	33,745,761	78,636,509	36,757	98,919	28.9	-0.174	E6PHBHT2
		P3HB4HB	68,415,798	40,296,848	216,456,195	134,016	288,620	30.6	-0.186	G7P4BHT2
	biodegradable polyesters	PBAT	49,902,238	39,109,600	277,380,367	176,551	386,366	24.5	-0.153	A8EcoflexT2
		PBSA	26,759,090	19,069,594	131,516,182	101,022	190,834	33.7	-0.176	B8PBSAT2
		PCL	47,562,104	38,754,135	130,034,674	89,264	182,400	27.1	-0.191	B9PCLT2
		PLLA	46,614,676	17,231,920	323,371,796	289,439	512,562	25.4	-0.147	C9PLLAT2
	common	PET	45,856,870	20,149,972	310,724,855	250,833	467,488	26.2	-0.162	C5PETT2
		PE	41,269,208	28,513,142	230,071,511	146,652	316,531	27.4	-0.170	G8LDPET2
		PS	43,209,890	21,667,334	238,667,503	159,189	332,956	23.9	-0.148	G9PST2
		PP	48,753,482	30,915,649	265,738,740	189,617	380,015	27.4	-0.171	F9PPT2
AMN13	PHAs	PHB	59,261,140	56,306,024	54,568,968	28,911	71,994	16.3	-0.138	T50P3HB
		PHBV	55,746,868	53,340,234	50,844,397	28,164	67,380	5.6	-0.117	T52PHBV
		PHBH	58,798,184	55,879,575	42,382,667	22,033	54,512	7.9	-0.119	T54PHBH
		P3HB4HB	47,133,656	44,650,207	58,660,684	34,295	78,979	13.1	-0.137	T57P4BH
	biodegradable polyesters	PBAT	33,480,472	31,661,521	68,301,009	29,361	85,169	20.8	-0.158	T58Ecoflex
		PBSA	35,331,670	30,658,999	113,836,609	53,037	141,832	24.4	-0.166	T60PBSA
		PCL	42,166,594	39,883,021	106,293,006	34,185	121,200	20.5	-0.156	T61PCL
		PLLA	53,718,346	48,084,846	137,676,553	76,392	180,573	23.2	-0.168	T62PLLA
	common	PET	36,198,300	32,212,918	112,326,015	73,290	158,052	22.8	-0.164	T702PET
		PE	49,501,666	43,122,330	134,868,147	68,119	169,977	23.0	-0.162	T68LDPE
		PS	71,211,024	59,147,875	145,104,539	84,889	188,597	22.3	-0.161	T69PS
		PP	30,950,364	26,949,100	130,150,162	57,328	158,668	24.0	-0.165	T67PP
BHT		WaterBHT*	123,819,272	50,970,849	397,329,399	344,455	632,827	32.6	-0.160	waterBHTc
		WaterBHT	113,954,372	48,916,098	397,509,359	335,541	624,781	32.9	-0.159	waterBHT
	sediment	BHT1cm*	100,868,178	10,293,915	221,857,546	265,699	411,503	39.3	-0.156	BHTw1cm
		BHT1cm	93,981,822	7,916,358	179,845,622	221,439	314,965	41.3	-0.153	BHT1cm
		BHT3cm	94,759,232	9,456,227	199,380,741	245,949	347,230	41.4	-0.159	BHT3cm
		BHT5cm	103,470,332	8,565,139	177,906,659	226,980	315,743	41.8	-0.161	BHT5cm
		BHT10cm	103,820,422	37,877,736	355,801,248	344,162	599,320	57.4	-0.182	BHT10cm

Supplementary Table 9 | Minimum information of metagenome-assembled genome (MiMAG) and GBDBtk taxonomy of MAGs.

Site_Plastic_MAG ID	GBDBtk taxonomy					G+C content	Genome size (bp)	N50 of scaffolds (bp)	No. contigs	No. scaffolds	CheckM		rRNAmmer			tRNA-scan	
	Phylum	Class	Order	family	genus						Completeness (%)	Contamination (%)	5s rRNA	16s rRNA	23s rRNA	No. tRNA	No. of AA missing in tRNAs
BHT04_PHB_gam2	Proteobacteria	Gammaproteobacteria	Enterobacterales	Alteromonadaceae	**Colwellia**	37.2%	3,890,228	11,196	700	641	95.1	5.0	3	1	1	58	0
BHT04_PHB_gam1	Proteobacteria	Gammaproteobacteria	Pseudomonadales	Cellvibrionaceae	**Agarilytica**	44.6%	5,302,014	129,448	112	98	99.1	1.4	1	1	1	41	1
AMN13_PHB_gam1	Proteobacteria	Gammaproteobacteria	Pseudomonadales	Cellvibrionaceae	**Agarilytica**	44.7%	5,420,529	80,306	143	125	99.1	2.1	0	0	0	39	1
BHT14_PHBH_gam1	Proteobacteria	Gammaproteobacteria	**QZLD01**	QZLD01	QZLE01	39.2%	3,108,749	264,084	26	26	93.7	1.6	1	1	1	35	0
PJM12_PHB_gam1	Proteobacteria	Gammaproteobacteria	**QZLD01**	QZLD01	QZLE01	38.9%	3,154,396	257,928	50	46	94.1	1.7	0	0	0	33	1
PJM12_PHB_gam2	Proteobacteria	Gammaproteobacteria	**QZLD01**	QZLD01	QZLE01	38.6%	3,263,329	107,260	80	58	92.3	1.7	0	0	0	32	1
BHT04_PCL_gam1	Proteobacteria	Gammaproteobacteria	Granulosicoccales	**Granulosicoccaceae**		57.1%	6,319,541	23,423	688	642	95.3	6.6	2	1	1	40	0
PJM02_PBSA_gam1	Proteobacteria	Gammaproteobacteria	Pseudomonadales	**Cellvibrionaceae**		46.7%	4,307,390	716,172	18	14	98.6	1.4	0	1	1	37	0
BHT04_PCL_gam2	Proteobacteria	Gammaproteobacteria	Pseudomonadales	Pseudomonadaceae	**Pseudomonas_D**	56.5%	3,889,687	1,039,964	27	23	100.0	1.0	1	1	1	48	0
BHT14_PBSA_del1	Desulfobacterota	Desulfobulbia	Desulfobulbales	Desulfocapsaceae		45.1%	4,989,220	63,495	202	165	97.3	1.2	1	1	1	38	1
BHT14_PCL_del1	Desulfobacterota	Desulfobacteria	Desulfobacterales	Desulfobacteraceae	**Desulfobacula**	39.2%	4,465,880	30,302	327	311	95.6	3.3	2	1	1	41	1
PJM12_PCL_del1	Desulfobacterota	Desulfobacteria	Desulfobacterales	Desulfobacteraceae		42.3%	5,169,573	166,481	83	75	99.0	0.7	1	1	1	41	1
BHT04_PCL_alp1	Proteobacteria	Alphaproteobacteria	Caulobacterales	Hyphomonadaceae	**Hyphomonas**	54.2%	3,277,584	404,467	10	10	98.7	2.2	1	1	1	36	0
BHT04_PBSA_alp1	Proteobacteria	Alphaproteobacteria	Rhodobacterales	Rhodobacteraceae	Marinosulfonomonas	54.0%	3,767,685	224,990	45	43	99.7	0.3	1	1	1	40	0
BHT04_PBAT_alp2	Proteobacteria	Alphaproteobacteria	Rhodobacterales	Rhodobacteraceae	Sulfitobacter	52.9%	4,833,856	220,187	64	61	99.4	1.5	1	1	0	39	0
AMN13_PHB_alp1	Proteobacteria	Alphaproteobacteria	Micavibrionales	**Micavibrionaceae**	UBA1672	44.3%	2,505,412	609,558	16	14	94.4	1.5	1	2	2	34	1
PJM02_PBAT_act1	**Actinobacteriota**	Acidimicrobiia	**Acidimicrobiales**	SZUA-35	SZUA-35	70.4%	4,238,728	27,009	376	296	97.3	1.3	1	1	1	36	1

Supplementary Table 10. Information of raw sequence data for 16S rRNA gene amplicon sequencing and metagenomic sequencing.

	DRA Submission (16S rRNA gene amplicon sequencing)					WGA Submission (metagenomic sequencing)			
Sample Name	BioProject	BioSample	DRA Submission	Experiment	Run	BioProject	BioSample	SRA Submission	Run
PHB_PJM02	PRJDB14280	SAMD00529542	DRA014821	DRX389282	DRR403672	PRJNA886482	SAMN31142920	SRP468711	SRR26541478
PHBV_PJM02	PRJDB14280	SAMD00529543	DRA014821	DRX389283	DRR403673	PRJNA886482	SAMN31142921	SRP468711	SRR26541477
PHBH_PJM02	PRJDB14280	SAMD00529544	DRA014821	DRX389284	DRR403674	PRJNA886482	SAMN31142922	SRP468711	SRR26541466
P3HB4HB_PJM02	PRJDB14280	SAMD00529545	DRA014821	DRX389285	DRR403675	PRJNA886482	SAMN31142923	SRP468711	SRR26541455
PBSA_PJM02	PRJDB14280	SAMD00529546	DRA014821	DRX389286	DRR403676	PRJNA886482	SAMN31142924	SRP468711	SRR26541444
PCL_PJM02	PRJDB14280	SAMD00529547	DRA014821	DRX389287	DRR403677	PRJNA886482	SAMN31142925	SRP468711	SRR26541433
PBAT_PJM02	PRJDB14280	SAMD00529548	DRA014821	DRX389288	DRR403678	PRJNA886482	SAMN31142926	SRP468711	SRR26541423
PLLA_PJM02	PRJDB14280	SAMD00529549	DRA014821	DRX389289	DRR403679	PRJNA886482	SAMN31142927	SRP468711	SRR26541422
PET_PJM02	PRJDB14280	SAMD00529550	DRA014821	DRX389290	DRR403680	PRJNA886482	SAMN31142928	SRP468711	SRR26541421
PE_PJM02	PRJDB14280	SAMD00529551	DRA014821	DRX389291	DRR403681	PRJNA886482	SAMN31142929	SRP468711	SRR26541420
PS_PJM02	PRJDB14280	SAMD00529552	DRA014821	DRX389292	DRR403682	PRJNA886482	SAMN31142930	SRP468711	SRR26541476
PP_PJM02	PRJDB14280	SAMD00529553	DRA014821	DRX389293	DRR403683	PRJNA886482	SAMN31142931	SRP468711	SRR26541475
CEA_PJM02	PRJDB14280	SAMD00529554	DRA014821	DRX389294	DRR403684	na	na	na	na
PRA_PJM02	PRJDB14280	SAMD00529555	DRA014821	DRX389295	DRR403685	na	na	na	na
MUA_PJM02	PRJDB14280	SAMD00529556	DRA014821	DRX389296	DRR403686	na	na	na	na
CETA_PJM02	PRJDB14280	SAMD00529557	DRA014821	DRX389297	DRR403687	na	na	na	na
PRTA_PJM02	PRJDB14280	SAMD00529558	DRA014821	DRX389298	DRR403688	na	na	na	na
MUTA_PJM02	PRJDB14280	SAMD00529559	DRA014821	DRX389299	DRR403689	na	na	na	na
PHB_PJM12	PRJDB14280	SAMD00529560	DRA014821	DRX389300	DRR403690	PRJNA886482	SAMN31142932	SRP468711	SRR26541474
PHBV_PJM12	PRJDB14280	SAMD00529561	DRA014821	DRX389301	DRR403691	PRJNA886482	SAMN31142933	SRP468711	SRR26541473
PHBH_PJM12	PRJDB14280	SAMD00529562	DRA014821	DRX389302	DRR403692	PRJNA886482	SAMN31142934	SRP468711	SRR26541472
P3HB4HB_PJM12	PRJDB14280	SAMD00529563	DRA014821	DRX389303	DRR403693	PRJNA886482	SAMN31142935	SRP468711	SRR26541471
PBSA_PJM12	PRJDB14280	SAMD00529564	DRA014821	DRX389304	DRR403694	PRJNA886482	SAMN31142936	SRP468711	SRR26541470
PCL_PJM12	PRJDB14280	SAMD00529565	DRA014821	DRX389305	DRR403695	PRJNA886482	SAMN31142937	SRP468711	SRR26541469
PBAT_PJM12	PRJDB14280	SAMD00529566	DRA014821	DRX389306	DRR403696	PRJNA886482	SAMN31142938	SRP468711	SRR26541468
PLLA_PJM12	PRJDB14280	SAMD00529567	DRA014821	DRX389307	DRR403697	PRJNA886482	SAMN31142939	SRP468711	SRR26541467
PE_PJM12	PRJDB14280	SAMD00529568	DRA014821	DRX389308	DRR403698	PRJNA886482	SAMN31142940	SRP468711	SRR26541465
PS_PJM12	PRJDB14280	SAMD00529569	DRA014821	DRX389309	DRR403699	PRJNA886482	SAMN31142941	SRP468711	SRR26541464
PP_PJM12	PRJDB14280	SAMD00529570	DRA014821	DRX389310	DRR403700	PRJNA886482	SAMN31142942	SRP468711	SRR26541463
CE_PJM12	PRJDB14280	SAMD00529571	DRA014821	DRX389311	DRR403701	na	na	na	na
PR_PJM12	PRJDB14280	SAMD00529572	DRA014821	DRX389312	DRR403702	na	na	na	na
MU_PJM12	PRJDB14280	SAMD00529573	DRA014821	DRX389313	DRR403703	na	na	na	na
CEA_PJM12	PRJDB14280	SAMD00529574	DRA014821	DRX389314	DRR403704	na	na	na	na
PRA_PJM12	PRJDB14280	SAMD00529575	DRA014821	DRX389315	DRR403705	na	na	na	na
MUA_PJM12	PRJDB14280	SAMD00529576	DRA014821	DRX389316	DRR403706	na	na	na	na
CETA_PJM12	PRJDB14280	SAMD00529577	DRA014821	DRX389317	DRR403707	na	na	na	na
PRTA_PJM12	PRJDB14280	SAMD00529578	DRA014821	DRX389318	DRR403708	na	na	na	na
MUTA_PJM12	PRJDB14280	SAMD00529579	DRA014821	DRX389319	DRR403709	na	na	na	na
PHB_BHT04	PRJDB14280	SAMD00529580	DRA014821	DRX389320	DRR403710	PRJNA886482	SAMN31142943	SRP468711	SRR26541462
PHBV_BHT04	PRJDB14280	SAMD00529581	DRA014821	DRX389321	DRR403711	PRJNA886482	SAMN31142944	SRP468711	SRR26541461
PHBH_BHT04	PRJDB14280	SAMD00529582	DRA014821	DRX389322	DRR403712	PRJNA886482	SAMN31142945	SRP468711	SRR26541460
P3HB4HB_BHT04	PRJDB14280	SAMD00529583	DRA014821	DRX389323	DRR403713	PRJNA886482	SAMN31142946	SRP468711	SRR26541459
PBSA_BHT04	PRJDB14280	SAMD00529584	DRA014821	DRX389324	DRR403714	PRJNA886482	SAMN31142947	SRP468711	SRR26541458
PCL_BHT04	PRJDB14280	SAMD00529585	DRA014821	DRX389325	DRR403715	PRJNA886482	SAMN31142948	SRP468711	SRR26541457
PBAT_BHT04	PRJDB14280	SAMD00529586	DRA014821	DRX389326	DRR403716	PRJNA886482	SAMN31142949	SRP468711	SRR26541456
PET_BHT04	PRJDB14280	SAMD00529587	DRA014821	DRX389327	DRR403717	PRJNA886482	SAMN31142950	SRP468711	SRR26541454
PLLA_BHT04	PRJDB14280	SAMD00529588	DRA014821	DRX389328	DRR403718	PRJNA886482	SAMN31142951	SRP468711	SRR26541453
PE_BHT04	PRJDB14280	SAMD00529589	DRA014821	DRX389329	DRR403719	PRJNA886482	SAMN31142952	SRP468711	SRR26541452
PS_BHT04	PRJDB14280	SAMD00529590	DRA014821	DRX389330	DRR403720	PRJNA886482	SAMN31142953	SRP468711	SRR26541451
PP_BHT04	PRJDB14280	SAMD00529591	DRA014821	DRX389331	DRR403721	PRJNA886482	SAMN31142954	SRP468711	SRR26541450
CE_BHT04	PRJDB14280	SAMD00529592	DRA014821	DRX389332	DRR403722	na	na	na	na
PR_BHT04	PRJDB14280	SAMD00529593	DRA014821	DRX389333	DRR403723	na	na	na	na
MU_BHT04	PRJDB14280	SAMD00529594	DRA014821	DRX389334	DRR403724	na	na	na	na
CEA_BHT04	PRJDB14280	SAMD00529595	DRA014821	DRX389335	DRR403725	na	na	na	na
PRA_BHT04	PRJDB14280	SAMD00529596	DRA014821	DRX389336	DRR403726	na	na	na	na
MUA_BHT04	PRJDB14280	SAMD00529597	DRA014821	DRX389337	DRR403727	na	na	na	na
CETA_BHT04	PRJDB14280	SAMD00529598	DRA014821	DRX389338	DRR403728	na	na	na	na
PRTA_BHT04	PRJDB14280	SAMD00529599	DRA014821	DRX389339	DRR403729	na	na	na	na
MUTA_BHT04	PRJDB14280	SAMD00529600	DRA014821	DRX389340	DRR403730	na	na	na	na
PHB_BHT14	PRJDB14280	SAMD00529601	DRA014821	DRX389341	DRR403731	PRJNA886482	SAMN31142955	SRP468711	SRR26541449
PHBV_BHT14	PRJDB14280	SAMD00529602	DRA014821	DRX389342	DRR403732	PRJNA886482	SAMN31142956	SRP468711	SRR26541448
PHBH_BHT14	PRJDB14280	SAMD00529603	DRA014821	DRX389343	DRR403733	PRJNA886482	SAMN31142957	SRP468711	SRR26541447
P3HB4HB_BHT14	PRJDB14280	SAMD00529604	DRA014821	DRX389344	DRR403734	PRJNA886482	SAMN31142958	SRP468711	SRR26541446
PBSA_BHT14	PRJDB14280	SAMD00529605	DRA014821	DRX389345	DRR403735	PRJNA886482	SAMN31142959	SRP468711	SRR26541445
PCL_BHT14	PRJDB14280	SAMD00529606	DRA014821	DRX389346	DRR403736	PRJNA886482	SAMN31142960	SRP468711	SRR26541443
PBAT_BHT14	PRJDB14280	SAMD00529607	DRA014821	DRX389347	DRR403737	PRJNA886482	SAMN31142961	SRP468711	SRR26541442
PLLA_BHT14	PRJDB14280	SAMD00529608	DRA014821	DRX389348	DRR403738	PRJNA886482	SAMN31142962	SRP468711	SRR26541441
PET_BHT14	PRJDB14280	SAMD00529609	DRA014821	DRX389349	DRR403739	PRJNA886482	SAMN31142963	SRP468711	SRR26541440
PE_BHT14	PRJDB14280	SAMD00529610	DRA014821	DRX389350	DRR403740	PRJNA886482	SAMN31142964	SRP468711	SRR26541439
PS_BHT14	PRJDB14280	SAMD00529611	DRA014821	DRX389351	DRR403741	PRJNA886482	SAMN31142965	SRP468711	SRR26541438
PP_BHT14	PRJDB14280	SAMD00529612	DRA014821	DRX389352	DRR403742	PRJNA886482	SAMN31142966	SRP468711	SRR26541437
CE_BHT14	PRJDB14280	SAMD00529613	DRA014821	DRX389353	DRR403743	na	na	na	na
PR_BHT14	PRJDB14280	SAMD00529614	DRA014821	DRX389354	DRR403744	na	na	na	na
MU_BHT14	PRJDB14280	SAMD00529615	DRA014821	DRX389355	DRR403745	na	na	na	na
CEA_BHT14	PRJDB14280	SAMD00529616	DRA014821	DRX389356	DRR403746	na	na	na	na
PRA_BHT14	PRJDB14280	SAMD00529617	DRA014821	DRX389357	DRR403747	na	na	na	na
MUA_BHT14	PRJDB14280	SAMD00529618	DRA014821	DRX389358	DRR403748	na	na	na	na
CETA_BHT14	PRJDB14280	SAMD00529619	DRA014821	DRX389359	DRR403749	na	na	na	na
PRTA_BHT14	PRJDB14280	SAMD00529620	DRA014821	DRX389360	DRR403750	na	na	na	na
MUTA_BHT14	PRJDB14280	SAMD00529621	DRA014821	DRX389361	DRR403751	na	na	na	na
PHB_AMN13	PRJDB14280	SAMD00529622	DRA014821	DRX389362	DRR403752	PRJNA886482	SAMN31142967	SRP468711	SRR26541436
PHBV_AMN13	PRJDB14280	SAMD00529623	DRA014821	DRX389363	DRR403753	PRJNA886482	SAMN31142968	SRP468711	SRR26541435
PHBH_AMN13	PRJDB14280	SAMD00529624	DRA014821	DRX389364	DRR403754	PRJNA886482	SAMN31142969	SRP468711	SRR26541434
P3HB4HB_AMN13	PRJDB14280	SAMD00529625	DRA014821	DRX389365	DRR403755	PRJNA886482	SAMN31142970	SRP468711	SRR26541432
PBSA_AMN13	PRJDB14280	SAMD00529626	DRA014821	DRX389366	DRR403756	PRJNA886482	SAMN31142971	SRP468711	SRR26541431
PCL_AMN13	PRJDB14280	SAMD00529627	DRA014821	DRX389367	DRR403757	PRJNA886482	SAMN31142972	SRP468711	SRR26541430
PBAT_AMN13	PRJDB14280	SAMD00529628	DRA014821	DRX389368	DRR403758	PRJNA886482	SAMN31142973	SRP468711	SRR26541429
PLLA_AMN13	PRJDB14280	SAMD00529629	DRA014821	DRX389369	DRR403759	PRJNA886482	SAMN31142974	SRP468711	SRR26541428
PET_AMN13	PRJDB14280	SAMD00529630	DRA014821	DRX389370	DRR403760	PRJNA886482	SAMN31142975	SRP468711	SRR26541427
PE_AMN13	PRJDB14280	SAMD00529631	DRA014821	DRX389371	DRR403761	PRJNA886482	SAMN31142976	SRP468711	SRR26541426
PS_AMN13	PRJDB14280	SAMD00529632	DRA014821	DRX389372	DRR403762	PRJNA886482	SAMN31142977	SRP468711	SRR26541425
PP_AMN13	PRJDB14280	SAMD00529633	DRA014821	DRX389373	DRR403763	PRJNA886482	SAMN31142978	SRP468711	SRR26541424
CE_AMN13	PRJDB14280	SAMD00529634	DRA014821	DRX389374	DRR403764	na	na	na	na
PR_AMN13	PRJDB14280	SAMD00529635	DRA014821	DRX389375	DRR403765	na	na	na	na
MU_AMN13	PRJDB14280	SAMD00529636	DRA014821	DRX389376	DRR403766	na	na	na	na
CEA_AMN13	PRJDB14280	SAMD00529637	DRA014821	DRX389377	DRR403767	na	na	na	na
PRA_AMN13	PRJDB14280	SAMD00529638	DRA014821	DRX389378	DRR403768	na	na	na	na
MUA_AMN13	PRJDB14280	SAMD00529639	DRA014821	DRX389379	DRR403769	na	na	na	na
CETA_AMN13	PRJDB14280	SAMD00529640	DRA014821	DRX389380	DRR403770	na	na	na	na
PRTA_AMN13	PRJDB14280	SAMD00529641	DRA014821	DRX389381	DRR403771	na	na	na	na
MUTA_AMN13	PRJDB14280	SAMD00529642	DRA014821	DRX389382	DRR403772	na	na	na	na
Water_BHTp	na	na	na	na	na	PRJNA886482	SAMN37935294	SRP468711	SRR26549052
Water_BHT	na	na	na	na	na	PRJNA886482	SAMN37935295	SRP468711	SRR26549051
Sediment1cm_BHTp	na	na	na	na	na	PRJNA886482	SAMN37935296	SRP468711	SRR26549050
Sediment1cm_BHT	na	na	na	na	na	PRJNA886482	SAMN37935297	SRP468711	SRR26549049
Sediment3cm_BHT	na	na	na	na	na	PRJNA886482	SAMN37935298	SRP468711	SRR26549048
Sediment5cm_BHT	na	na	na	na	na	PRJNA886482	SAMN37935299	SRP468711	SRR26549047
Sediment10cm_BHT	na	na	na	na	na	PRJNA886482	SAMN37935300	SRP468711	SRR26549046
