# Supplementary material for: Microbial decomposition of biodegradable plastics on the deep-sea floor
Source: Nat Commun. 2024 Jan 26;15:568. doi: 10.1038/s41467-023-44368-8 (PMC10817984; doi:10.1038/s41467-023-44368-8)
Supplement: Supplementary file 3 — Description of Additional Supplementary Files [file 41467_2023_44368_MOESM3_ESM.pdf]

Description of Additional Supplementary Files:

Supplementary Data 1: Biodegradation test for film samples.

Supplementary Data 2: BOD test for PHBH and cellulose.

Supplementary Data 3: Biodegradation test for injection-molded samples.

Supplementary Data 4: Relative frequency of ASV within each plastisphere and the taxonomic assignment.

Supplementary Data 5: Taxonomic classification of RpsC sequences within the metagenomes of plastispheres established at the sea.

Supplementary Data 6: RpsC-based relative frequency (%) based on genus level taxa within plastispheres established at the sea.

Supplementary Data 7: Raw Data. RpsC-based relative frequency (%) based on genus level taxa within plastispheres established at the sea.

Supplementary Data 8: Taxonomic classification of RpsC sequences within the metagenomes of plastispheres established at the sea.

Supplementary Data 9: List of MAG-associated scaffolds and coverages of the scaffolds mapped by different metagenomic reads.
