# Supplementary material for: Microbial decomposition of biodegradable plastics on the deep-sea floor
Source: Nat Commun. 2024 Jan 26;15:568. doi: 10.1038/s41467-023-44368-8 (PMC10817984; doi:10.1038/s41467-023-44368-8)
Supplement: Supplementary file 5 — Reporting Summary [file 41467_2023_44368_MOESM5_ESM.pdf]

nature portfolio

Corresponding author(s): Tadahisa Iwata

Last updated by author(s): Nov 12, 2023

Reporting Summary

Nature Portfolio wishes to improve the reproducibility of the work that we publish. This form provides structure for consistency and transparency in reporting. For further information on Nature Portfolio policies, see our Editorial Policies and the Editorial Policy Checklist.

Statistics

For all statistical analyses, confirm that the following items are present in the figure legend, table legend, main text, or Methods section.

n/a	Confirmed	
☐	☒	The exact sample size (*n*) for each experimental group/condition, given as a discrete number and unit of measurement
☐	☒	A statement on whether measurements were taken from distinct samples or whether the same sample was measured repeatedly
☒	☐	The statistical test(s) used AND whether they are one- or two-sided<br>*Only common tests should be described solely by name; describe more complex techniques in the Methods section.*
☒	☐	A description of all covariates tested
☒	☐	A description of any assumptions or corrections, such as tests of normality and adjustment for multiple comparisons
☐	☒	A full description of the statistical parameters including central tendency (e.g. means) or other basic estimates (e.g. regression coefficient) AND variation (e.g. standard deviation) or associated estimates of uncertainty (e.g. confidence intervals)
☒	☐	For null hypothesis testing, the test statistic (e.g. *F*, *t*, *r*) with confidence intervals, effect sizes, degrees of freedom and *P* value noted<br>*Give P values as exact values whenever suitable.*
☒	☐	For Bayesian analysis, information on the choice of priors and Markov chain Monte Carlo settings
☒	☐	For hierarchical and complex designs, identification of the appropriate level for tests and full reporting of outcomes
☒	☐	Estimates of effect sizes (e.g. Cohen's *d*, Pearson's *r*), indicating how they were calculated

Our web collection on statistics for biologists contains articles on many of the points above.

Software and code

Policy information about availability of computer code

Data collection	DNA extraction for 16S rRNA sequencing: NanoDrop 2000/2000c version 1.5, SkanIt RE for Varioskan Flash version 2.4.5<br>Extraction of metagenome-assembled genomes: CheckM v1.1.0, tRNAscan-SE v.2.0, RNAmmer version 1.2, GTDB-tk v1.3.0
Data analysis	Visualization of weight loss and thickness reduction data, BOD, on-the-figure captioning, and maps: Microsoft Office 365 (Word, Excel, Power Point), OriginPro 2018, GeoMapApp version 3.6.14, Adobe Illustrator 2023<br>Read processing for 16S rRNA sequencing: QIIME2 pipeline version qiime2-2021.4, BLASTN version 2.12<br>DNA analysis for 16S rRNA sequencing: MEGA 11 software version 11.0.10, R v4.0.2, qiime2R version 0.99.6, phyloseq version 1.34.0, metagMisc version 0.0.4, ggplot2 version 3.3.5, ggrepel version 0.9.1<br>Read processing and estimation of environmental parameters: CLC Genomics Workbench version 20.0, CLC Genome Finishing Module version 20.0, MetaGeneMark server, GhostKOALA version 2.0, calc.gc.pl from Multi-Metagenome pipeline<br>RpsC-based microbial community analysis: KEGG Automatic Annotation Server (KAAS) ver April 3, 2015, XLSTAT ECOLOGY ver 2020.4.1.1014<br>Functional annotation of esterase: TMHMM server version 2.0, ESTHER database version May 27, 2020<br>Global distribution of dominant microbes: DADA2, Qiime2 v. 2020.82, Ocean Data View 5.6.03

For manuscripts utilizing custom algorithms or software that are central to the research but not yet described in published literature, software must be made available to editors and reviewers. We strongly encourage code deposition in a community repository (e.g. GitHub). See the Nature Portfolio guidelines for submitting code & software for further information.

Data

Policy information about availability of data

All manuscripts must include a data availability statement. This statement should provide the following information, where applicable:

- Accession codes, unique identifiers, or web links for publicly available datasets
- A description of any restrictions on data availability
- For clinical datasets or third party data, please ensure that the statement adheres to our policy

All raw sequences of 16S rRNA amplicon sequencing have been deposited at DDBJ (DNA Data Bank of Japan) Sequence Read Archive (DRA) under accession number DRA014821 (https://ddbj.nig.ac.jp/resource/sra-submission/DRA014821) in BioProject PRJDB14280 (https://ddbj.nig.ac.jp/resource/bioproject/PRJDB14280). All raw sequences of metagenomic sequencing have been deposited at NCBI Sequence Read Archive (SRA) under accession number SRP468711 (https://www.ncbi.nlm.nih.gov/sra/?term=SRP468711) in BioProject PRJNA886482 (https://www.ncbi.nlm.nih.gov/bioproject/?term=PRJNA886482). The detailed information of the sequence reads was summarized in Supplementary Table 10. The scaffold sequences of each MAG have been deposited at DDBJ/EMBL/GenBank under BioProject PRJNA886482 as biosamples SAMN38046932- SAMN38046948. The data supporting the findings presented in Figure 2-4 are available within the paper and the Supplementary Information files and Source Data.

Research involving human participants, their data, or biological material

Policy information about studies with human participants or human data. See also policy information about sex, gender (identity/presentation), and sexual orientation and race, ethnicity and racism.

Reporting on sex and gender	N/A
Reporting on race, ethnicity, or other socially relevant groupings	N/A
Population characteristics	N/A
Recruitment	N/A
Ethics oversight	N/A

Note that full information on the approval of the study protocol must also be provided in the manuscript.

Field-specific reporting

Please select the one below that is the best fit for your research. If you are not sure, read the appropriate sections before making your selection.

☐ Life sciences ☐ Behavioural & social sciences ☒ Ecological, evolutionary & environmental sciences

For a reference copy of the document with all sections, see nature.com/documents/nr-reporting-summary-flat.pdf

Ecological, evolutionary & environmental sciences study design

All studies must disclose on these points even when the disclosure is negative.

Study description	This study evaluates the extent of biodegradability of "biodegradable plastics" processed into injection molded components and melt-pressed films, at five different deep-sea environments across Japan with two different time periods in each location. In addition, microorganisms attached to the surface of the plastics are also analyzed. The analysis was performed with n=5, but in some cases where sampling in large numbers is difficult, n=2. The degradation data were carried out in a nested design where the influence of variables associated with the location such as depth, microbiome was analysed on the outcome of the degradation of the plastic samples.
Research sample	A total of 21 different plastics were analyzed in this study. Out of the 21 samples, 4 were of microbial polyesters class, (polyhydroxyalkanoates), 4 biodegradable polyesters, 9 different kinds of polysaccharide ester derivatives. To compare the degradability of these plastics with conventional, commercial, plastics, polyethylene, polypropylene, polystyrene, polyethylene terephthalate were considered as standards. With respect to metagenomic analyses, micro-organisms with PHB depolymerase genes (for PHA samples) and cutinases/polyesterases (other biodegradable plastics) in their genomes were identified and analyzed in this work. Specifically, Colwellia, Agarilytica, and Micavibrio correlated with PHA plastispheres in aerobic conditions, while the order QZLD01 correlated with anaerobic conditions. In terms of other biodegradable plastics, Profundibacter, Hyphomonas and Desulfobacula were each found to have secreted polyesterases and cutinases and were thus analysed further.
Sampling strategy	No statistical methods for sample size calculation were performed. The degradability of the plastics was determined based on the data from the n=5 samples. The average values were reported with its standard deviation. Typical biodegradability experiments reported in the past have had a sample size of n=3 or n=5, which was adopted in this study as well. Since, the study is of an exploratory nature with multiple practical constraints, the authors deduced that a n=5, would be able to provide reliable results while keeping a balance with the execution difficulty.

Data collection	The samples were prepared/fabricated by the authors of this work. Every set of samples was then assigned a unique serial number. The weight and dimensions of the samples before and after submerging in the deep-sea were measured by the laboratory's postdoctoral researchers and students in a single-blind system. Efforts were taken to keep the human error to a minimum by establishing measurement protocols.<br>Access to the submerging sites was done using JAMSTEC regulated research vessels, "Yokosuka" and "Kaimei". The deployment and recovery of deep-sea samples were conducted by the HOV human occupied vehicle (HOV) Shinkai 6500 or remotely operated vehicles (ROVs; Hyper-Dolphin and Kaimei-ROV), except in the abyssal plain around Minamitorishima Island, where the free-fall-type deep-sea observatory lander system Edokko Mark-I was employed.
Timing and spatial scale	Data collection was carried out between 2019 and 2021 in different locations distanced about 50km-2200km from the port of JAMSTEC, along the Japanese coast in the northern Pacific ocean. Initially, the degradation rate of plastics in the deep sea was expected to be slow, so the collection of installed samples was set to approximately once every six months. The exact installation and recovery dates are mentioned in Table 1 in the main article. Since the installation sites were in the deep-sea, at times recovery had to be rescheduled by a couple of days due to rough weather and other related factors beyond the control of the authors.
Data exclusions	All data were used for the analysis.
Reproducibility	Since the experiment could not be repeated many times in the same location and over the same period of time, all installation samples were first prepared in multiple sets, and installed in the chosen deep-sea location at once. The samples were then recovered, after six months and one year, and analyzed for changes of weight, and dimensions with respect to time. This method reduces the error compared to the preparation of the sample multiple times. In addition, since the analysis was performed with n=5 for each sample and changes over time were also observed, reproducibility seems to be ensured.
Randomization	The weight-measured plastic samples were put into chambers that would be submerged into the deep sea. All the samples placed in each chamber were chosen randomly.
Blinding	All the prepared samples for the experiment were assigned a unique serial number. The laboratory's postdoctoral researchers and students then did the weight and dimension measurements of the samples in a single-blind measurement.

Did the study involve field work? ☒ Yes ☐ No

Field work, collection and transport

Field conditions	All plastics were placed on the deep-sea floors at five different locations with different environmental conditions.<br>BHT: Bathyal hydrocarbon seepage Off Hatsushima Island (depth=855m, Temp.=3.6oC, dissolved oxygen (DO)=1.8mg/L)<br>BMS: Bathyal seafloor Off Misaki Port (depth=757m, Temp.=4.4 oC, dissolved oxygen (DO)=2.1mg/L)<br>BMJ: Bathyal hydrothermal vent in Myojin Knoll (depth=1,292m, Temp.=4.6 oC, dissolved oxygen (DO)=2.1mg/L)<br>AKR: Abyssal Plain near Kuroshio extension observatory (depth=5,503m, Temp.=1.6 oC, dissolved oxygen (DO)=5.2mg/L)<br>AMN: Abyssal plain around Minamitorishima Island (depth=5,552m, Temp.=1.5 oC, dissolved oxygen (DO)=5.0mg/L)
Location	The environmental biodegradation tests were performed on the deep-sea floor at five sites: (1) three bathyal sites [the bathyal seafloor off Misaki port, BMS (35°4.2'N, 139°32.5'E, at a depth of 757 m below sea level); hydrocarbon seepage off Hatsushima Island, BHT (35°0.9'N, 139°13.3'E, at a depth of 855 m below sea level); and a hydrothermal vent off Myojin Knoll, BMJ (32°6.3'N, 139°52.2'E, at a depth of 1,292 m below sea level)], and<br>(2) two abyssal sites [the abyssal plain near Kuroshio extension observatory, AKR (32°34.8'N, 143°46.1'E, at a depth of 5,503 m below sea level), and the abyssal plain around Minamitorishima Island, AMN (22°59.9N, 154°24.5E, at a depth of 5,552 m below sea level)].<br><br>As a reference site that is close to large cities and rivers, the same tests were also performed in a coastal environment [port of JAMSTEC Yokosuka Headquarters, PJM (35°19.2'N, 139°39.0'E, at a depth of 2–6 m)].
Access & import/export	Since the experiments did not include any materials that were restricted by the Japanese national or international laws, no permits were required for the use. All the samples used in the study or materials required to produce the samples are in fact commercially available. Access to the deep-sea submerging sites was provided by JAMSTEC regulated submersibles and support vessels, "Kairei", "Yokosuka" and "RV Kaimei", voyage numbers:<br>1. KM20-09 (14-11-2020 to 22-11-2020) Kaimei, Kaimei-ROV<br>2. KM21-E02 (2-2-2021 to 6-2-2021) RV Kaimei, Kaimei-ROV<br>3. KR21-04C (03-04-2021 to 21-04-2021) Kairei, Edokko Mark-I<br>4. YK21-08C (10-5-2021 to 29-5-2021) Yokosuka, Shinkai6500<br>5. YK21-18C (28-9-2021 to 17-10-2021) Yokosuka, Shinkai6500
Disturbance	None

Reporting for specific materials, systems and methods

We require information from authors about some types of materials, experimental systems and methods used in many studies. Here, indicate whether each material, system or method listed is relevant to your study. If you are not sure if a list item applies to your research, read the appropriate section before selecting a response.

Materials & experimental systems

n/a	Involved in the study
☒	☐ Antibodies
☒	☐ Eukaryotic cell lines
☒	☐ Palaeontology and archaeology
☒	☐ Animals and other organisms
☒	☐ Clinical data
☒	☐ Dual use research of concern
☒	☐ Plants

Methods

n/a	Involved in the study
☒	☐ ChIP-seq
☒	☐ Flow cytometry
☒	☐ MRI-based neuroimaging
